# Myeloid cell interferon responses correlate with clearance of SARS-CoV-2

**DOI:** 10.1038/s41467-022-28315-7

**Published:** 2022-02-03

**Authors:** Dhiraj K. Singh, Ekaterina Aladyeva, Shibali Das, Bindu Singh, Ekaterina Esaulova, Amanda Swain, Mushtaq Ahmed, Journey Cole, Chivonne Moodley, Smriti Mehra, Larry S. Schlesinger, Maxim N. Artyomov, Shabaana A. Khader, Deepak Kaushal

**Affiliations:** 1 Southwest National Primate Research Center, Texas Biomedical Research Institute, San Antonio, TX 78245 USA; 2 Department of Medicine, Washington University in St. Louis, St. Louis, MO 63110 USA; 3 Department of Molecular Microbiology, Washington University in St. Louis, St. Louis, MO 63110 USA; 4 Tulane National Primate Research Center, Tulane University School of Medicine, Covington, LA 70433 USA

**Keywords:** Viral infection, Innate immunity

## Abstract

Emergence of mutant SARS-CoV-2 strains associated with an increased risk of COVID-19-related death necessitates better understanding of the early viral dynamics, host responses and immunopathology. Single cell RNAseq (scRNAseq) allows for the study of individual cells, uncovering heterogeneous and variable responses to environment, infection and inflammation. While studies have reported immune profiling using scRNAseq in terminal human COVID-19 patients, performing longitudinal immune cell dynamics in humans is challenging. Macaques are a suitable model of SARS-CoV-2 infection. Our longitudinal scRNAseq of bronchoalveolar lavage (BAL) cell suspensions from young rhesus macaques infected with SARS-CoV-2 (*n* = 6) demonstrates dynamic changes in transcriptional landscape 3 days post- SARS-CoV-2-infection (3dpi; peak viremia), relative to 14-17dpi (recovery phase) and pre-infection (baseline) showing accumulation of distinct populations of both macrophages and T-lymphocytes expressing strong interferon-driven inflammatory gene signature at 3dpi. Type I interferon response is induced in the plasmacytoid dendritic cells with appearance of a distinct HLADR^+^CD68^+^CD163^+^SIGLEC1^+^ macrophage population exhibiting higher angiotensin-converting enzyme 2 (ACE2) expression. These macrophages are significantly enriched in the lungs of macaques at 3dpi and harbor SARS-CoV-2 while expressing a strong interferon-driven innate anti-viral gene signature. The accumulation of these responses correlated with decline in viremia and recovery.

## Abstract (web summary)

To gain insights into the early immune dynamics of transcriptional changes during SARS-CoV-2 infection, Singh et al. provide longitudinal scRNA-Seq of the broncho-alveolar compartment of SARS-CoV-2 infected rhesus macaques. They observe an accumulation of a distinct macrophage population that possesses an interferon-driven innate anti-viral gene signature early during infection that correlates with viral clearance.

## Introduction

The underlying immune mechanisms that drive disease versus protection during the Coronavirus disease 2019 (COVID-19) are not well understood. Analysis of system-wide transcriptomic responses can be extremely useful in identifying features of protection and pinpoint the host immune processes involved in the control of infection and drivers of pathology^1,2^. Transcriptional changes in cells in the broncho-alveolar lavage (BAL) and peripheral blood mononuclear cells (PBMCs) of COVID-19 patients show distinct host inflammatory cytokine profiles, suggesting that excessive cytokine release is associated with COVID-19 pathogenesis^3^. However, analyses were conducted using end-point samples in patients, and it is possible that the excessive cytokine storm at that time is a representation of an exacerbated viral infection and associated immune dysregulation. We recently developed a nonhuman primate (NHP) model of SARS-CoV-2 infection^4^, where NHPs develop signs of COVID-19 disease including characteristic ground-glass opacities in lungs, coinciding with a cytokine storm and a myeloid cell influx, followed by clearance of the virus and recovery^4^. This model has been used extensively for the evaluation of both therapeutics^5^ and vaccines^6^ against COVID-19. Using RNAseq, we showed that Interferon (IFN) signaling, neutrophil degranulation, and innate immune pathways were significantly induced in the SARS-CoV-2-infected lungs of NHPs, while pathways associated with collagen formation were downregulated^7^. Since these animals controlled infection naturally, our results highlight the importance of early innate immune responses and cytokine signaling, particularly Type I IFN signaling, in protecting against COVID-19. One limitation of the above study was that it was conducted in terminal lung samples and thus may not represent the dynamic changes that occur immediately after infection. Furthermore, system-wide transcriptomics was studied using bulk-RNAseq, thus averaging the overall contributions of various cell types and pathways at play.

The use of single-cell technologies such as single-cell RNA-sequencing (scRNAseq) allows for unbiased and significantly more in-depth profiling of immune cell populations in animal models and humans in both healthy and diseased states. Because scRNAseq can define the transcriptomic heterogeneity of a complex community of cells and assign unbiased identity classifications to cell populations, it is optimally suited for the study of complex inflammatory states such as the one engendered by SARS-CoV-2 infection. ScRNAseq has recently identified initial cellular targets of SARS-CoV-2 infection in model organisms^8–11^ and patients^12–17^ and characterized peripheral and local immune responses in severe COVID-19^18^, which is associated with a cytokine storm and increased neutrophil accumulation. However, the human studies have mostly been performed in peripheral blood samples^18^, BAL^12^, and tissues^19^ from a limited number of moderate or severe COVID-19 patients within limited age ranges. To overcome the limitations associated with longitudinal early immune profiling in human subjects and to get more in-depth understanding of the early dynamics of transcriptional changes during COVID-19, we characterized the transcriptional signatures at the single-cell level in the broncho-alveolar compartment of rhesus macaques at pre-infection collected 7 days before infection (−7 dpi), at acute phase of SARS-CoV-2 infection (3 dpi) and endpoint of the study (14–17 dpi). Thus, the immune landscape in the broncho-alveolar compartment of SARS-CoV-2 infected rhesus macaques serves as a surrogate of early immune dynamics of protective immune responses in lungs after SARS-CoV-2 infection. Here we show the appearance of distinct macrophage and T-lymphocyte populations exhibiting IFN-driven inflammatory gene signatures at 3dpi (acute COVID-19). The IFN responsive macrophage populations upregulate ACE2 expression and are infected by SARS-CoV-2. Further analysis of upregulated genes in the macrophages reveal IFN-driven innate antiviral defense and negative regulation of viral genome replication, suggesting a prominent role of macrophage-driven innate immunity in the resolution of SARS-CoV-2 infection.

## Results

### Landscape of immune cells in the BAL of rhesus macaques during SARS-CoV-2 infection and recovery

**Fig. 1:**
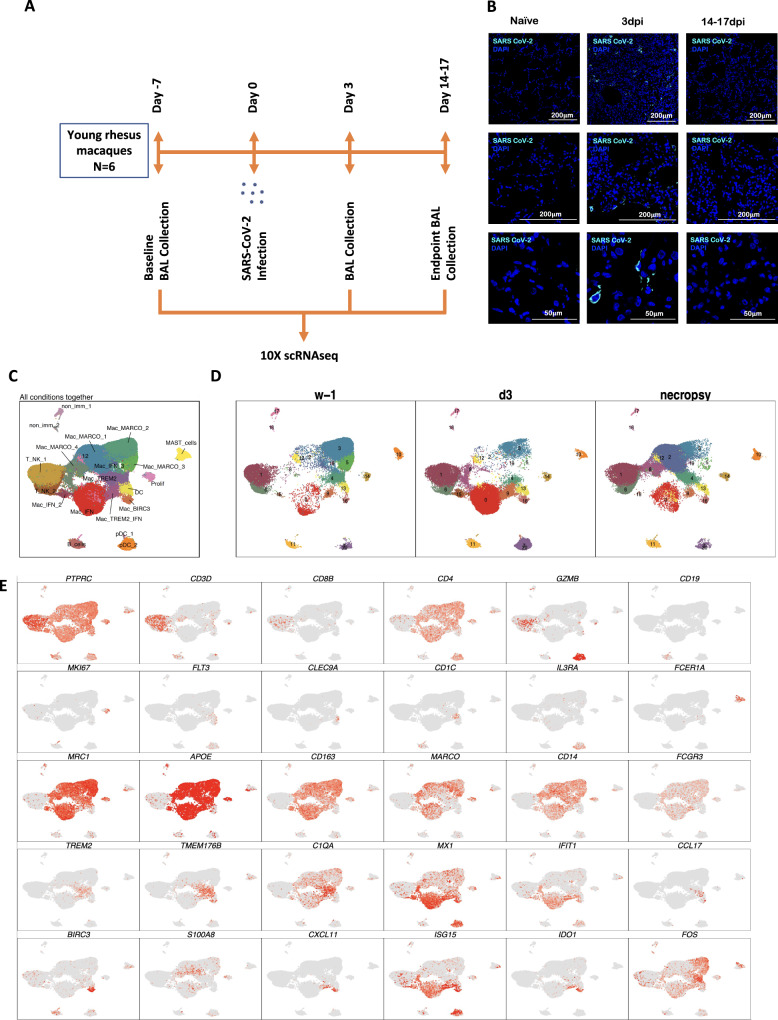
Immune landscape of BAL in SARS-CoV-2 infected macaques. Study outline of scRNAseq analysis of BAL cells from rhesus macaques infected with SARS-CoV-2. BAL single-cell suspensions from 6 young rhesus macaques infected with SARS-CoV-2 from pre-infection (-7dpi), 3dpi and endpoint (14–17dpi) were subjected to scRNAseq (**A**). Immunofluorescence confocal images of the lungs stained with nucleocapsid (N)-specific antibodies (turquoise) and 4,6-diamidino-2-phenylindole (DAPI) (blue). Shown are the images at 10x, 20x, and 63x magnification from naive lungs (uninfected) as well as lungs infected with SARS-CoV-2 at Day 3 and Day 14 post-infection (**B**). UMAP plots of cells from all scRNAseq samples together, colored according to cluster classification (**C**) or respective timepoints (**D**). **E** UMAP plots with the expression of markers, characterizing main immune populations. *n* = 6.

To understand the early immune responses generated by SARS-CoV-2 in the NHP model of COVID-19, we analyzed cryopreserved single cells isolated from BAL of young rhesus macaques infected by SARS-CoV-2^4^ at the following time points: 7 days before infection (−7 dpi), three days post-infection (3 dpi), endpoint (14−17 dpi) (Fig. 1A). These time points were validated to represent distinct phases of viral dynamics in vivo covering baseline, the peak of acute viral infection (3 dpi), and at the time that infection had resolved (endpoint) (Fig. 1B)^4^.

**Fig. 2:**
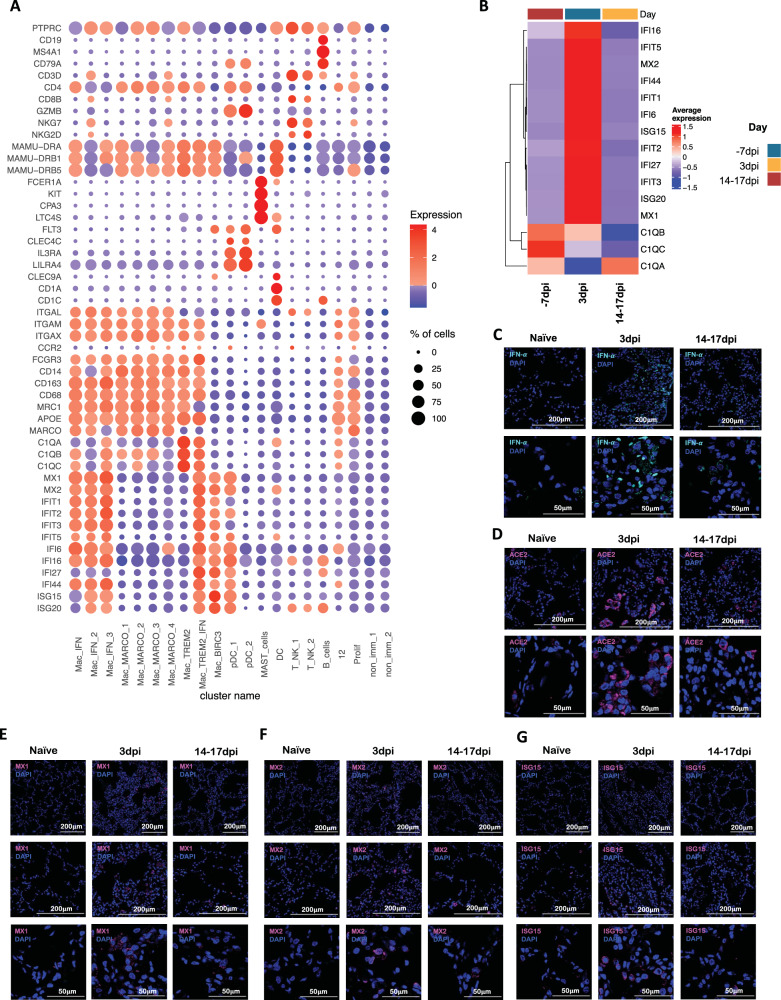
SARS-CoV-2 infection induces IFN responsive gene signature in rhesus macaques. **A** Bubble plot showing the fold change of genes in identified cell clusters and the fraction of cells expressing the gene of interest. **B** Heatmap of key interferon responsive genes at different timepoints. Confocal images validating in vivo expression of IFN-α (turquoise) (**C**), ACE2 (magenta) (**D**), MX1 (magenta) (**E**), MX2 (magenta) (**F**) and ISG15 (magenta) (**G**) with DAPI (blue) in the lung sections of Naive rhesus macaques and SARS-CoV-2 infected lungs at 3dpi and 14–17 dpi.

We subjected single cells isolated from BAL of the SARS-CoV-2 infected rhesus macaques to 3′ 10x Genomics based scRNAseq processing and analysis pipeline with rigorous QC threshold (Supplementary Fig. 1) at −7dpi (*n* = 6), 3 dpi (*n* = 6), and 14–17 dpi (*n* = 6). Sequencing yielded a total of 170078 cells ranging from 1543-16608 cells per sample. The mean number of cells per sample in −7 dpi was 8484, 3 dpi was 9840 and 14–17 dpi was 10,021. The majority of the cells were immunocytes (Fig. 1C) distributed across all time points (Fig. 1D) and animals (Fig. 1C, Supplementary Fig. 2). Consistent with the prior reports on the cellular composition of BAL in rhesus macaques, myeloid cells were most abundant comprising 77% of the total cells whereas lymphoid cells represented 22% of cells at all time points. The populations were homogeneously distributed across all animals and at all time points (Supplementary Fig. 2b). We identified 19 distinct cell clusters representing a variety of cell types based on expression of canonical genes- T cells: cluster of differentiation (CD) 3D^+^; natural killer (NK) cells: killer cell lectin-like receptor C3(KLRC3)^+^, Granzyme B (GZMB)^+^; B cells: CD19^+^, CD20/membrane spanning 4-domains A1 (MS4A1)^+^, CD79A^+^; macrophages: CD68^+^, CD163^+^) dendritic cells (DC): FMS-like tyrosine kinase 3 (FLT3)^+^; conventional DCs (cDC): CD1c^+^; plasmacytoid DCs (pDC): (CD123/interleukin (IL) 3 receptor subunit alpha (IL3RA)^+^, CD303/ C-type lectin domain family 4 member C (CLEC4C)^+^, leukocyte immunoglobulin-like receptor A4 (LILRA4)^+^ and mast cells: (CD117/ KIT proto-oncogene (KIT)^+^, Fc fragment of IgE receptor 1a (FCER1A)^+^, carboxypeptidase A3 (CPA3)^+^ (Fig. 1C, E, Fig. 2A, Supplementary Fig. 3). Day 3 was distinguished by the appearance of cell populations expressing a distinct IFN responsive gene signature comprising of transcripts for MX dynamin-like GTPase (MX) 1, MX2, interferon-induced protein with tetratricopeptide repeats (IFIT) 1, IFIT2, IFIT3, IFIT5, IFN-α inducible protein^20^ 6, IFI16, IFI44, interferon-stimulated gene (ISG) 15, HECT and RLD domain containing E3 ubiquitin protein ligase (HERC) 5, sialic acid binding Ig like lectin (SIGLEC) 1, 2′-5′-oligoadenylate synthetase (OAS) 1, OAS2, OAS3 (Fig. 2B, Supplementary Fig. 4). The most prominent population (Cluster 0) appearing at 3dpi was IFN responsive macrophages and was annotated Mac_IFN. Although IFN-α and ACE2 transcripts were not abundantly present in the scRNAseq dataset, confocal analysis showed strong upregulation of IFN-α and ACE-2 in the lungs of macaques on 3dpi compared to healthy or 14–17dpi (Fig. 2C, D). Confocal analyses also demonstrated higher expression of IFN responsive transcripts like MX1 (Fig. 2E), MX2 (Fig. 2F), and ISG15 (Fig. 2G) in lung tissues isolated from rhesus macaques at 3dpi when compared to 14–17dpi and healthy macaques.

As described previously, SARS-CoV-2 vRNA levels in BAL were detected in 5/6 macaques at 3 dpi by RT-qPCR. Virtually no BAL vRNA was detected at the endpoint suggesting that the rhesus macaques effectively cleared the virus from the BAL compartment^4^. vRNA in Nasal Swabs (NS) could be detected in only four animals at day 3, while all animals recorded vRNA at Day 9, and only 3 at the endpoints^4^. vRNA was detected in the lungs of 3 macaques at necropsy (14–17 dpi) while no SARS CoV-2 subgenomic RNA (correlate for infectious/replicating virus) was detected in any rhesus macaque lung at endpoints^4^. No vRNA was detected in any plasma samples or randomly selected urine samples. Based on vRNA persistence in the lungs of immunocompetent young macaques and the absence of replicative virus in our cohort and published reports from other independent groups^21–25^, we conclude that macaques efficiently control SARS-CoV-2 infection over the two-week study period. Consistent with the peak viremia at 3 dpi in BAL, the chest x-ray scores were also found to peak around 3dpi and subsided thereafter. However, tissue pathology was observed in the lung at endpoints suggesting that persistent viral antigens triggered a sustained immune/inflammatory response. Despite the absence of replicating virus in lungs at endpoints, pathological observations were found as shown by histological analyses^4^.

Our previous immunological analyses of BAL cells by flow cytometry had revealed a massive infiltration of immunocytes mainly comprised of T cells, interstitial macrophages, neutrophils, and plasmacytoid Dendritic Cells^4^. The appearance of these populations also correlated strongly with the viral loads^4^. When combined with the previously reported findings, our new results using scRNA-seq based deep cellular phenotyping analysis establish the robust influx of myeloid cells and induction of a fully functional IFN driven innate immune response in macrophages against SARS-CoV-2 in the lungs of rhesus macaques. Our scRNA-seq based deep cellular phenotyping analysis clearly establishes the induction of a robust and targeted innate immune response mainly driven by macrophages as opposed to dysregulated immune responses against SARS-CoV-2 in the early phase of infection.

### Myeloid bronchoalveolar landscape

**Fig. 3:**
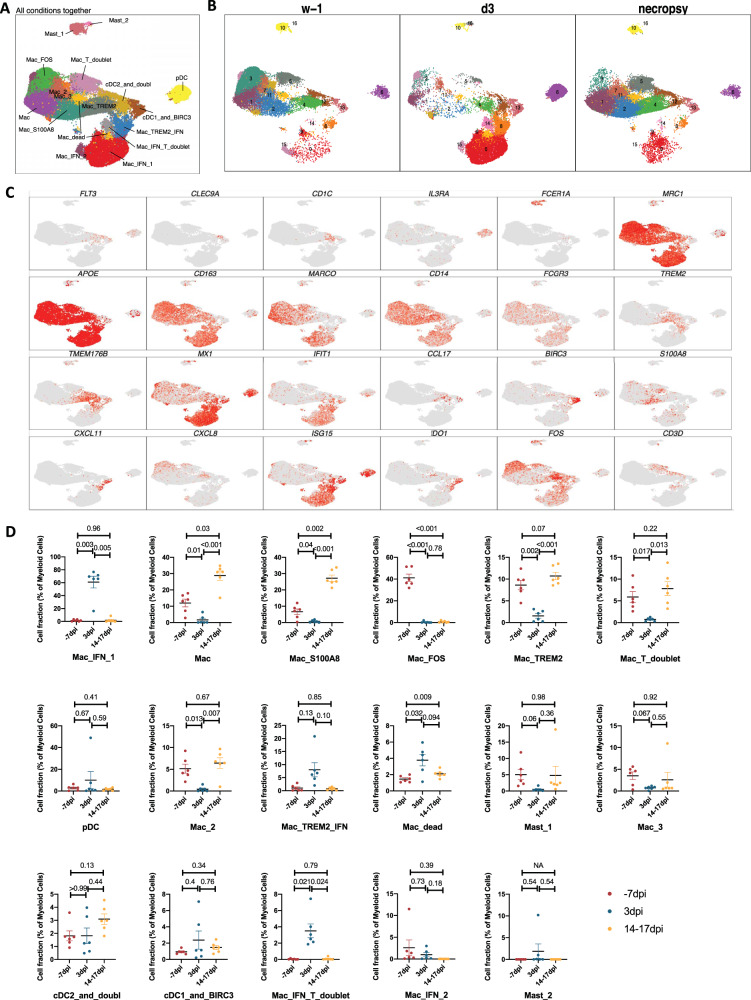
Myeloid single-cell landscape in SARS-CoV-2 infected macaques. BAL myeloid cell dynamics in macaques infected with SARS-CoV-2 by scRNA-seq demonstrate the presence of IFN response in pDCs and IFN-responsive macrophage compartments. **A** UMAP plot of myeloid cells from all scRNA-seq samples together, colored according to (**A**) cluster classification or respective (**B**) timepoints. **C** UMAP plots with the expression of markers, characterizing main myeloid populations in macaques. **D** Cell proportion of each cluster per condition. *n* = 6 macaques. Data are presented as mean ± standard error of the mean (SEM). Source data are provided as a source data file.

A total of 129280 myeloid cells were analyzed across all time points which were distributed into 17 distinct clusters across the 3 timepoints studied (Fig. 3A, B, Supplementary Fig. 5A). The populations were homogeneously distributed across all animals and time points (Supplementary Fig. 5B). We noted a distinct cluster alignment of all myeloid populations based on key myeloid phenotype markers (Fig. 3C, D) that differed between different phases of disease (Fig. 3D).

**Fig. 4:**
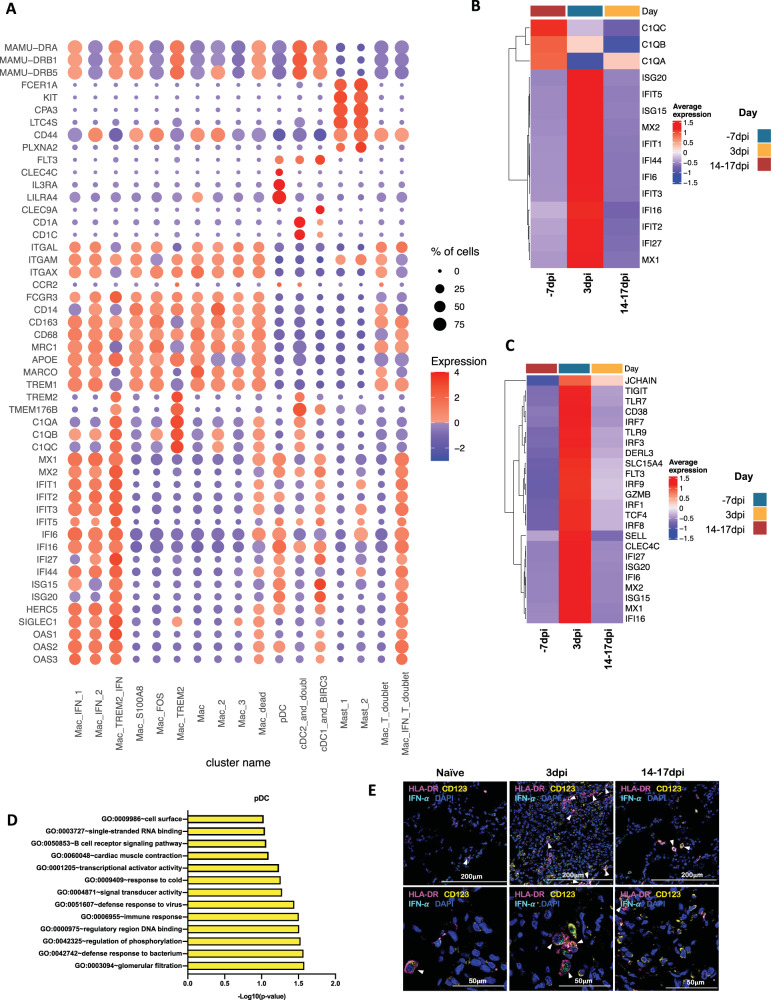
Macrophages and pDCs are the dominant cells driving Type I IFN response in the lungs of SARS-CoV-2 infected macaques. **A** Bubble plot showing the fold change of genes in identified myeloid cell clusters and the fraction of cells expressing the gene of interest. **B** Heatmap of key interferon responsive genes at different timepoints in macrophage clusters. **C** Heatmap of key interferon responsive genes at different timepoints in plasmacytoid dendritic cells sub-clusters. **D** GO pathways enriched in upregulated genes in pDCs. **E** Multilabel immunofluorescence confocal images validating in vivo expression of IFN-α (turquoise) in pDCs marked by HLA-DR (magenta) and CD123 (yellow) in Naive Rhesus macaque lungs as well as at Day 3 and Day 14 post-infection with SARS CoV-2.

As expected, due to limitation of the 10x Genomics scRNAseq pipeline to detect neutrophils^26^, this population was not included in our analysis. As reported earlier in various NHP studies^4,27,28^, the BAL landscape mostly comprised of macrophages, which are distributed into alveolar (CD206^+^) or interstitial (CD206^−^) phenotypes^4,28^. Our prior scRNAseq analysis using the 10x platform for single cells isolated from the lungs of rhesus macaques with tuberculosis had identified novel macrophage phenotypes exhibiting distinct TREM2 and IFN-responsive gene signatures^26^. Here, we found 3 distinct IFN-responsive macrophages populations which were predominantly present on 3dpi (Fig. 4A, B, Supplementary Figs. 6, 7), one of which also expressed high levels of triggering receptor expressed on myeloid cells (TREM) 2 gene expression (Fig. 4A, Supplementary Fig. 6). For reference, we annotated the most abundant IFN-responsive macrophage population as Mac_IFN_1, the second IFN responsive macrophage population was annotated Mac_IFN_2 and the third IFN responsive macrophages with a TREM2 expression module were annotated Mac_TREM2_IFN (Fig. 4A, Supplementary Fig. 6).

pDCs are considered the chief drivers and source of the Type I IFN signature. Our prior data suggested a significant influx of pDCs in the BAL compartment and lungs of SARS-CoV-2 infected macaques^4^. The pDC cluster in our current scRNAseq data was identified by expression of classic pDC markers like IL3RA/CD123, CLEC4C, and transcription factor (TCF) 4 (Fig. 4A, Supplementary Fig. 6). This cluster was only modestly increased at 3dpi. However, the pDC cluster exhibited a significant induction of the genes associated with the innate response to viral pathogens like Toll-like receptors (TLR)7, TLR9 etc., and induction of genes involved in the type I IFN response, e.g., interferon regulatory factor (IRF) 1, IRF3, IRF7, IRF8, IRF9, derlin (DERL) 3, solute carrier family 15 member 4 (SLC15A4). In addition, the expression of an IFN responsive transcriptional signature (MX1, MX2, ISG15, ISG20, IFI6, IFI16, IFI27) was also significantly elevated in this pDC cluster (Fig. 4C, D, Supplementary Fig. 7). Multilabel confocal microscopy analysis validated the higher expression of IFN-α by pDCs in lung tissues isolated from infected macaques at 3dpi (Fig. 4E, Supplementary Figs. 8, 9A) when compared to 14-17dpi and healthy macaques (Fig. 4E, Supplementary Figs. 8, 9A).

**Fig. 5:**
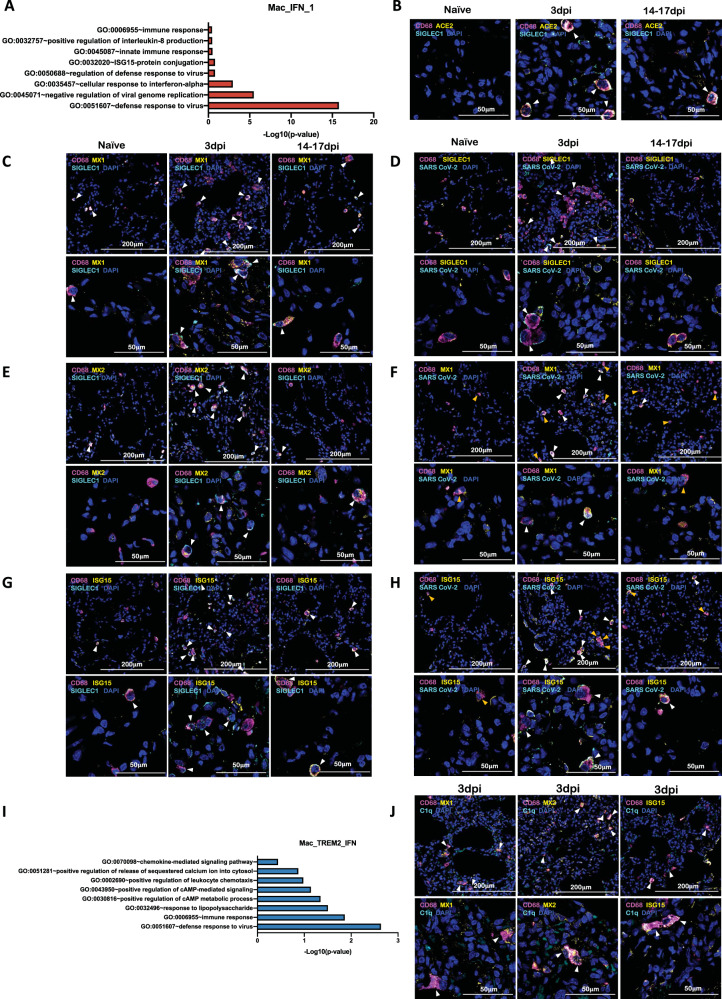
IFN induced viral defense response in lung macrophages of SARS-CoV-2 infected macaques. **A** GO pathways enriched in upregulated genes in Mac_IFN_1 subcluster. Multilabel immunofluorescence confocal images validating in vivo expression of (**B**) ACE2 (yellow) and SIGLEC1 (turquoise), and (**C**) MX1 (yellow) and SIGLEC1 (turquoise) in macrophages (magenta), (**D**) CD68 (magenta) and SIGLEC1 (yellow) positive macrophages harboring SARS CoV-2 (turquoise), (**E**) Macrophages (magenta) expressing MX2 (yellow) and SIGLEC1 (turquoise), (**F**) MX1 (yellow) positive macrophages (magenta) with SARS CoV-2 (turquoise), (**G**) Macrophages (magenta) expressing ISG15 (yellow) and SIGLEC1 (turquoise), (**H**) SARS CoV-2 (turquoise) harbored in ISG15 (yellow) expressing macrophages (magenta) in lungs of Naive and 3 and 14 days post-infection of SARS CoV-2 infected macaques. Nuclei stained with DAPI are shown in blue. White arrows represent macrophages expressing ACE2 and SIGLEC1 in (**B**); MX1 and SIGLEC1 in (**C**); MX2 and SIGLEC1 in (**E**); ISG15 and SIGLEC1 in (**G**). In (**D**), (**F**), and (**H**), white arrows mark the presence of SARS CoV-2 in macrophages expressing SIGLEC1, MX1, and ISG15 respectively; whereas, orange arrows are used to mark SIGLEC1, MX1, and ISG15 expressing macrophages with no SARS CoV-2. **I** GO pathways enriched in upregulated genes in Mac_TREM2_IFN subcluster. **J** Multilabel confocal immunofluorescence images validating in vivo expression of MX1, MX2, and ISG15 (shown in yellow) in TREM2 macrophages in lungs of SARS-CoV-2 infected macaques at 3dpi.

Among macrophage subclusters, mac_IFN_1 was the most abundant population found in 3dpi BAL samples, comprising 70 percent of the myeloid cells; this population was completely absent in pre-infection and during resolution at endpoint. IFN-responsive gene signature was strongly upregulated in this population and the key genes which were most differentially upregulated in this population were MX1, MX2, IFIT1, IFIT2, IFIT3, IFIT5, IFI6, IFI16, IFI44, ISG15, HERC5, SIGLEC1, OAS1, OAS2, OAS3 etc. (Figs. 3D, 4A, Supplementary Fig. 6). MX1 encodes a guanosine triphosphate (GTP)-metabolizing protein called IFN-induced GTP-binding protein Mx1 which is induced by Type I and Type II IFNs, antagonizes the replication process of several RNA and DNA viruses and participates in the cellular antiviral responses^29–32^. MX2, a paralog of MX1, is another IFN-induced GTP-binding protein that induces innate antiviral immune responses. IFIT genes encode IFN-induced antiviral proteins which act as inhibitors of cellular as well as viral processes, cell migration, proliferation, signaling, and viral replication^33,34^. IFI6 is one of the earliest identified IFN induced genes encoding the IFN-α -inducible protein 6 which has been shown to exert antiviral activity towards viruses by inhibiting the EGFR signaling pathway^35–38^. IFI16 gene encodes the Interferon Gamma Inducible protein 16 which is involved in the sensing of intracellular DNA and inducing death of virus-infected cells^39–42^. IFI44 encodes the Interferon-Induced protein 44 which is induced by Type 1 but not Type II IFNs and is reported to suppress viral transcription^43,44^. IFIT genes encode for the IFIT proteins (Interferon Induced proteins with Tetratricopeptide repeats) which confer antiviral state in a cell by either directly binding to the viral RNA or by binding to eukaryotic initiation factor 3 (eIF3) and preventing eIF3 from initiating viral translational processes^45–47^. All four classes of IFIT, i.e., IFIT1, IFIT2, IFIT3, and IFIT5 were upregulated in the mac_IFN_1 population (Fig. 4A). ISG15 also called Ubiquitin-like protein ISG15 is an early mediator of signaling induced by Type I IFNs and elicits innate immune response to viral infections by conjugation/ISGylation of its targets like MX and IFIT^48–51^. OAS encodes IFN-induced, dsRNA-activated antiviral enzyme which plays a critical role in cellular innate antiviral responses^52,53^. HERC5 is an E3 ligase for ISG15 conjugation which acts as a positive regulator of innate antiviral response in cells induced by IFNs and functions as part of the ISGylation machinery^54–56^. SIGLEC1 (CD169) is an IFN-inducible gene that acts as an endocytic receptor mediating clathrin-dependent endocytosis and has been reported to be upregulated in circulating monocytes in COVID-19 patients^57–60^. Gene set enrichment analysis (GSEA) analysis revealed defense response to virus, negative regulation of viral genome replication, response to IFN-α, and innate immune response as enriched gene ontology (GO) terms in this population (Fig. 5A).

SARS-CoV-2 infection of CD169^+^ macrophages has been reported in COVID-19^19,61^. To determine whether SARS-CoV-2 infects CD169^+^ macrophages in our study, lung tissues from SARS-CoV-2 infected rhesus macaques and healthy controls were stained for CD68, ACE2, SIGLEC1, MX1, MX2, ISG15, Complement component 1q (C1q) and SARS CoV-2 nucleocapsid antibody (Fig. 5B–H, J, Supplementary Figs. 9B, C, 10-17). Lung macrophages expressing SIGLEC1/CD169 were enriched and expressed high levels of ACE2 at 3dpi (Fig. 5B, Supplementary Fig. 10). Another independent study has established that human macrophages and monocytes can be infected by SARS-CoV-2 but the infection is abortive^62^. The macrophage population was further studied for detecting IFN responsive elements: MX1 (Fig. 5C, Supplementary Fig. 11), MX2 (Fig. 5E, Supplementary Fig. 13), ISG15 (Fig. 5G, Supplementary Fig. 15) and viral antigens (Fig. 5D, Supplementary Fig. 12; Fig. 5f, Supplementary Figs. 14; 5H, S16) and were found to abundantly harbor SARS-CoV-2 in vivo as shown by multicolor confocal staining for SARS-CoV-2 Nucleocapsid (NP) protein in lung sections. MX1/MX2/ISG15 staining with viral antigen clarified that the IFN-responsive signature was mostly restricted to macrophages harboring SARS-CoV-2, confirming an early IFN-driven innate immune response in lung macrophages.

The Mac_IFN_2 cluster bore a high degree of identity to the Mac_IFN_1 cluster, with one important difference—it expressed a comparatively higher expression of transcripts for C-X-C motif chemokine ligand. (CXCL) 8, IL1B and tumor necrosis factor (TNF) associated with nuclear factor-kappa B (NFKB) inhibitor zeta (NFKBIZ), NFKBIA, TNF-α-induced protein 3 (TNFAIP3) and activator protein (AP)1 signaling: Fos proto-oncogene^63^, Jun proto-oncogene (JUN), JunB proto-oncogene (JUNB).

We previously reported a novel cell cluster of alveolar macrophages abundantly found in the lungs of rhesus macaques showing an enriched expression of TREM2, transmembrane protein (TMEM) 176A/B and C1Q genes^26^. Our current data validated the presence of two clusters of macrophages showing TREM2 gene signatures, one of which expressed a strong IFN-responsive gene signature. Therefore, we annotated them as Mac_TREM2 and Mac_TREM2_IFN respectively. Mac_TREM2 cluster was abundantly present in the BAL from healthy macaques and switched to an IFN-responsive phenotype on 3dpi which was then restored at the endpoint (Fig. 3D). Mac_TREM2 cluster observed at pre-infection baseline was replenished to normal levels at endpoint (Fig. 3D) demonstrated enriched expression of transcripts for FOS, FosB proto-oncogene (FOSB), activating transcription factor (ATF) 3, Regulator Of G Protein Signaling^64^ 1, Aryl hydrocarbon receptor (AHR), NFKBIZ, BTG anti-proliferation factor (BTG) 2, early growth response (EGR) 1, lamin A/C (LMNA), RasGEF domain family member 1B (RASGEF) 1B and CD69 (Fig. 4A). The Mac_IFN_TREM2 cluster showed an upregulation of IFN-responsive gene signature comprising of SIGLEC1, MX, IFIT, IFI, ISG, and OAS genes (Fig. 4A), suggesting defense response to virus as significantly enriched gene set (Fig. 5I). Validation in lung sections of macaques confirmed the abundance of TREM2 macrophages with IFN-responsive phenotype on 3dpi (Fig. 5J, Supplementary Fig. 17).

Mac_FOS cluster was abundant in BAL at baseline and constituted ~40-50% of myeloid cells. However, this cluster was depleted at 3dpi and was not restored at the endpoint. Mac_FOS expressed higher transcripts for CXCL8 expression along with FOS, FOSB, NFKBIA, NFKBIZ AHR, lysozyme (LYZ), and CD69. GSEA revealed that this subset expressed an inflammatory and neutrophil chemotactic signature (Supplementary Fig. 18A), even in absence of infection in healthy macaques.

Mac_S100A8 cluster constituted 10% of the myeloid cells at pre-infection baseline, but was absent on 3dpi and abundantly present at the endpoint suggesting a potential role for these cells in post-acute COVID-19 pathology (Fig. 3D). The key transcripts upregulated in this cluster were S100 calcium binding protein (S100) A4, S100A6, S100A8, S100A9, cathelicidin antimicrobial peptide (CAMP), carboxypeptidase vitellogenic like (CPVL) (Fig. 4A) which represent innate inflammatory immune responses, neutrophil aggregation, and chemotaxis pathways (Supplementary Fig. 18B). Mac_S100A8 cluster has four genes significantly upregulated from the S100 family of genes that involve low molecular-weight proteins considered as potent damage-associated molecular pattern molecules (DAMPs). DAMPs are also called danger signals or alarmins as they serve as a warning sign for the innate immune system to alert ambient damage or infection. S100A8 protein also called calgranulin A forms a heterodimer with S100A9 protein called calgranulin B, to form a heterodimer called Calprotectin which stimulates T-lymphocyte chemotaxis by acting as a chemoattractant complex with peptidoglycan recognition protein 1 (PGLYRP1) that promotes lymphocyte migration via C-C chemokine receptor (CCR) 5/ C-X-C motif chemokine receptor (CXCR) 3 receptors^65,66^; neutrophil recruitment along with TLR4 and/or receptor for advanced glycation end products^67^ -mediated multiple inflammatory pathways^68,69^. Intracellular functions of S100A6 include regulation of several cellular functions, such as proliferation, apoptosis, cytoskeleton dynamics, response to different stress factors etc. When secreted into extracellular milieu it also induces RAGE (receptor for advanced glycation end-products) and integrin β1 mediated inflammatory responses^70^. S100A4 synergizes with vascular endothelial growth factor (VEGF) in a RAGE-dependent manner to promote endothelial cell migration by increasing KDR (kinase insert domain receptor)/vascular endothelial growth factor receptor 2 (VEGFR2) expression and MMP-9 activity^71^. S100A4 also plays a major role in high-density collagen deposition^72^.

Three other populations of macrophages lacking IFN signature (annotated Mac, Mac_2 & Mac_3) were present in healthy macaques at pre-infection timepoint. These populations were non-existent at 3dpi but replenished at the endpoints (Figs. 3D, 4A).

**Fig. 6:**
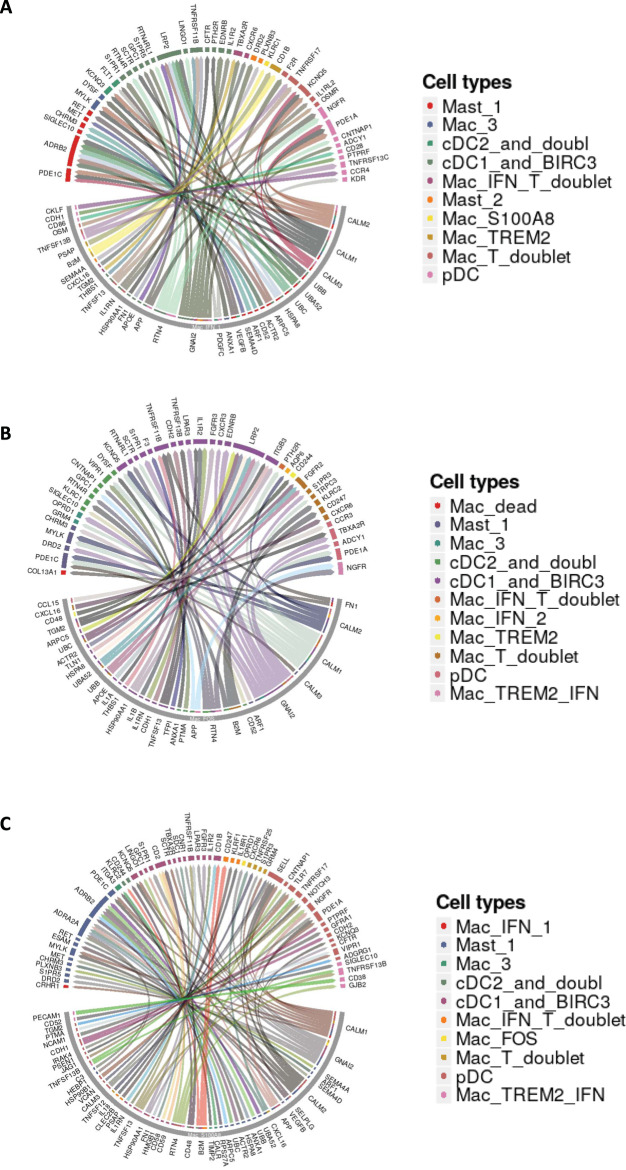
Macrophage interactome model in SARS-CoV-2 infected lungs. Macrophage-Immunocytes interactome. Circos plots showing the ligand-receptor interactions between the most abundant macrophage populations and different immune cells in the three conditions studies. **A** Circos plot depicting the interaction of Mac_IFN_1 with ambient immunocytes based on ligand-receptor transcript reads at 3 dpi. **B** Circos plot depicting the interaction of Mac_S100A8 with ambient immunocytes based on ligand-receptor transcript reads at 14–17 dpi. **C** Circos plot depicting the interaction of Mac_FOS with ambient immunocytes based on ligand-receptor transcript reads at −7 dpi.

To further understand the influence of macrophages during SARS-CoV-2 on other immunocytes, we analyzed the ligand-receptor interactions between the most abundant macrophage population and other immunocytes based on cell-specific transcripts expressed at different timepoints. The ligand-receptor interactions between Mac_IFN_1 and other immunocytes present at 3 dpi depicted as Circos plot (Fig. 6A) shows a prominent co-stimulatory potential of Mac_IFN_1 population on mast cells via adrenoceptor beta (ADRB) 2, SIGLEC10, cholinergic receptor muscarinic (CHRM) 3; cDCs via LDL receptor related protein (LRP) 2, TNF receptor superfamily member (TNFRSF) 11B, and pDCs via nerve growth factor receptor (NGFR), CD28. Mac_FOS showed prominent interaction with cDCs (Fig. 6B) and the Mac_S100A8 cluster with Mast cells, cDCs, pDCs in addition to Mac_TREM2_IFN (Fig. 6C).

### Lymphoid bronchoalveolar landscape

**Fig. 7:**
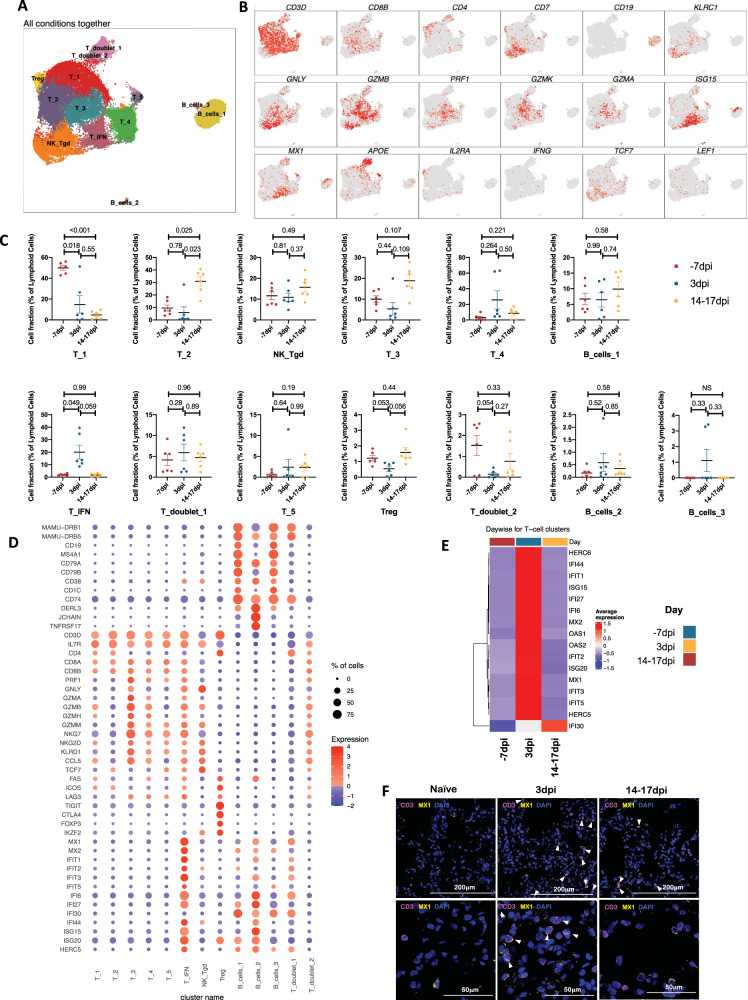
Lymphoid single-cell landscape in SARS-CoV-2 infected macaques. BAL lymphoid cell dynamics in macaques infected with SARS-CoV-2 by scRNA-seq demonstrate the presence of IFN responsive T cells. **A** UMAP plot of lymphoid cells from all scRNA-seq samples together, colored according to cluster classification. **B** UMAP plots with the expression of markers, characterizing main lymphoid populations in macaques. **C** Cell proportion of each cluster per condition. *n* = 6 macaques. Data are presented as mean ±  standard error of the mean (SEM). Source data are provided as a source data file. **D** Bubble plot showing the fold change of genes in identified lymphoid cell clusters and the fraction of cells expressing the gene of interest. **E** Heatmap of key interferon responsive genes at different timepoints in lymphoid sub-clusters. **F** Multilabel immunofluorescence confocal images validating in vivo expression of MX1 (yellow) in T-cells marked by CD3 (magenta) and nuclei (blue) in Naive as well as SARS CoV-2 infected lungs at 3 dpi and 14–17 dpi. White arrows represent T cells expressing MX1.

A total of 38160 lymphoid cells were analyzed across all timepoints which distributed into 13 distinct clusters (Fig. 7A, Supplementary Fig. 19a, b). The populations were homogenously distributed across all animals at each timepoint (Supplementary Fig. 19c). We noted distinctive cluster alignment of all lymphoid populations based on key lymphocyte phenotype markers (Fig. 7A, B) that differed between different phases of disease (Fig. 7C, D, Supplementary Fig. 20). The only distinct lymphocyte cluster found to be upregulated at 3dpi was a T cell cluster with IFN responsive gene signature spanning MX1, MX2, ISG15, ISG20, IFI27, IFI44, IFIT1, IFIT2, IFIT3, IFIT5, OAS1, OAS2, HERC5 HERC6 genes and was annotated T_IFN (Fig. 7D, E, Supplementary Figs. 20–21). Confocal analysis in lung sections validated the abundance of IFN responsive T cells at 3dpi in macaque lungs (Fig. 7F, Supplementary Figs. 9D, 22).

Regulatory T cells (T_Regs_) constituted <3% of the lymphoid population and expressing negative checkpoint regulators (NCR) like inducible costimulator (ICOS), lymphocyte activating (LAG) 3, T cell immunoreceptor with Ig and ITIM domains (TIGIT) etc. along with forkhead box P3 (FOXP3). When compared to pre-infection baseline T_Regs_ showed a slight decline at 3 dpi but were replenished at the endpoints. IFN-α can drive contraction of T_Regs_ while ISG15 can rescue T_Regs_ from Type I IFN induced contraction^73^. We observed high IFN-α concentrations at day 3, and relatively lower expression of ISG15. These results could explain the contraction of T_Regs_ at 3 dpi.

## Discussion

A thorough understanding of the host inflammatory responses during SARS-CoV-2 infection is needed both the identification of correlates of protection versus pathology. Such information is also critical in order to identify pathways that can be precisely modulated to limit inflammation without affecting protective mechanisms. During the acute phase of SARS-CoV-2 infection, macrophages were the most abundant immunocyte population that harbored vRNA in african green monkey lung cells^9^. Similarly, infected ferrets exhibited infiltration of monocyte-derived macrophages and induction of inflammatory responses in the bronchoalveolar lavage^10^. Similar induction of early innate defense responses in acute phase of COVID-19 at 3 dpi with resolution by 7 dpi in the lungs of rhesus macaques, has recently been reported^11^. Recognition of viral infections by innate immune sensors activates both the Type I and Type III IFN responses. Accumulating evidence has established that SARS-CoV-2 elicits weaker induction of type 1 IFNs when compared to other respiratory viruses^74^ and is marked by comparatively less responsive IL-1 and NLRP3 inflammasome pathways in early or non-severe COVID-19 patients^75^. Elevated levels of IFNs have been reported to correlate with and contribute to severe COVID-19^7,76–84^. It is possible that severe infection drives an uncontrolled expression of the Type I IFN response leading to pathology instead of viral containment. However, inborn errors of Type I IFN in COVID-19 patients have been associated with life-threatening conditions^78,79^. Similar life-threatening manifestation of COVID-19 has also been attributed to patients having autoantibodies against type I IFNs^84^. It is possible that delayed or inadequate IFN responses lead to inflammation-mediated damage during later phases of disease. Increased induction of early Type I IFN signaling pathways in SARS-CoV-2 infected macaques suggests a role for IFN signaling in protection rather than disease progression^4^. This is further supported by the increased induction of Type I IFN signaling in the cohort of young relative to geriatric macaques^7^, suggesting that IFN induction may be compromised in older or immunocompromised hosts^7,85^. Thus, it is not fully clear if Type I IFNs are protective or pathological in COVID-19^85^. This is an important paradox to resolve, as it could lead to better therapeutic approaches for COVID-19 as well as for long-term persistent COVID-19 sequelae. Elegant approaches are available to modulate the signaling of this pathway in macaques^86^. Our studies lay the foundation of Type I IFN depletion studies in this model to better understand the role of this pathway in the early control of SARS-CoV-2 infection and in limiting inflammation. Further testing the protective versus pathological roles of IFNs in different phases of COVID-19 in the macaque model with the availability of IFNAR blocking reagents should further clarify the specific role of IFN pathways in COVID-19.

Our results unequivocally show that in protected, immunocompetent hosts, SARS-CoV-2 infection is characterized by an acute inflammatory response leading to a myeloid cell influx into the lung compartment^4^. A key characteristic of this inflammatory response is a strong Type I IFN response^7^. Using state-of-the-art scRNAseq approach in longitudinal BAL samples, we now demonstrate that the robust Type I IFN response and related cytokine expression, observed in the airways of infected macaques is primarily mediated by myeloid cell subpopulations that are alveolar (206+) rather than interstitial (206−) in nature. In particular, macrophage subpopulations Mac_IFN_1 (206+), Mac_IFN_2 (206+) and Mac_TREM2_IFN (206−) subpopulations expressed high levels of IFN downstream genes both in magnitude and frequency (Fig. 2). Our results clearly show that induction of a robust IFN response in macrophages strongly correlates with viremia (Supplementary Fig. 23A, B) and subsequent clearance of SARS-CoV-2 from the airways of macaques (Supplementary Fig. 23C, D).

## Methods

### Macaques

No live Indian-origin rhesus macaques were used in this study. Samples obtained from young Rhesus macaques *(Macaca mulatta)* infected with 1.05 × 10^6^ pfu SARS-CoV-2 isolate USA-WA1/2020 (BEI Resources, NR-52281, Manassas, VA) using multiple routes (ocular, intranasal and intratracheal) enrolled in a previously described study^4^ were used for further analysis (Table S1). All procedures were approved by the Biohazard and Safety Committee and Institutional Animal Care and Use Committee of the Texas Biomedical Research Institute. The exposure stock was confirmed to be SARS-CoV-2 using deep sequencing and was identical to the published sequence (GenBank: MN985325) strain USA-WA1/2020 (BEI Resources, NR-52281).

### Isolation of BAL single cells from macaques

Single-cell suspensions from BAL obtained at different time points were collected as described earlier^4,87^ and cryopreserved in Cryostor-CS10 (Biolife Solutions, USA) at −70 °C and then used for downstream processing of scRNAseq.

### Single-cell RNA: library generation and sequencing

scRNAseq was done according to the manufacturer’s instructions (10x genomics) and as previously described^88^. Briefly, after quickly thawing the frozen BAL single-cell suspension in water bath, 2 × 10^6^ cells were taken for downstream processing. BAL single-cell suspensions were subjected to droplet-based massively parallel single-cell RNA sequencing using Chromium Single Cell 3′ (v3.1) Reagent Kit in the BSL-3 laboratory as per manufacturer’s instructions (10x Genomics). Briefly, cell suspensions were loaded at 1000 cells/μL with the aim to capture 10,000 cells/lane. The 10x Chromium Controller generated GEM droplets, where each cell was labeled with a specific barcode, and each transcript labeled with a unique molecular identifier^89^ during reverse transcription. The barcoded cDNA was isolated and removed from the BSL-3 space for library generation. The cDNA underwent 11 cycles of amplification, followed by fragmentation, end repair, A-tailing, adapter ligation, and sample index PCR as per the manufacturer’s instructions. Libraries were sequenced on a NovaSeq S4 (200 cycles) flow cell, targeting 30,000 read pairs/cell.

### Single-cell RNAseq: data processing

The Cell Ranger Single-Cell Software 3.0 available at 10x website was used to perform sample demultiplexing. We aligned resulting fastq files on mmul10 genome (Genebank, https://www.ncbi.nlm.nih.gov/assembly/GCF _003339765.1/), with the addition of Ensembl mmul8 mitochondrial genes for GTF file with cellranger count. For each sample, the recovered-cells parameter was set to 10,000 cells that we expected to recover for each individual library.

We used R package Seurat 3^90^ for downstream analysis of count matrixes that we got as output from cellranger count^88^. We filtered cells that (1) had more than 10% of mitochondrial gene content and^91^ had <363 detected genes. Data was log-normalized with a scale factor of 10^4^. The most variable genes were detected by the FindVariableFeatures function and used for subsequent analysis. Latent variables (number of UMI’s and mitochondrial content) were regressed out using a negative binomial model (function ScaleData). Principle component analysis^64^ was performed with RunPCA function. A UMAP dimensionality reduction was performed on the scaled matrix (with most variable genes only) using the first 20 PCA components to obtain a two-dimensional representation of the cell states. For clustering, we used the functions FindNeighbors (20 PCA) and FindClusters (resolution 0.5). Identified clusters were split into 2 cell groups: myeloid and lymphoid—and ran through re-clustering pipelines individually. For both cell subsets reclustering we performed clustering on the first 20 PCA components. We used clustering resolution 0.35 for myeloid and lymphoid cells reclustering. To identify marker genes, we used FindAllMarkers to compare clusters against all other clusters, and FindMarkers to compare selected clusters. For each cluster, only genes that were expressed in more than 15% of cells with at least 0.15-fold difference were considered. Heatmap representations were generated as described earlier with Phantasus software (https://artyomovlab.wustl.edu/phantasus/)^88^, using the mean expression of markers inside each cluster for each sample was used.

### Circos plots

Circos plots depicting possible cell interactions were created using SingleCellSignalR^92^.

### Immunohistochemistry and Confocal Imaging

To validate the findings of BAL single-cell sequencing, multilabel immuno-histochemistry was performed on Naive (four randomly selected lung lobes from four macaques) and SARS CoV-2 infected Rhesus macaque lungs at Day 3 (four randomly selected lung lobes from two macaques) and Day 14-17 (four randomly selected lung lobes from four macaques) post-infection as described^4^. The lung sections were stained for macrophages with anti-CD68 antibody, SIGLEC1 with anti-CD169 antibody, Mac_IFN_1 signature markers with anti-MX1, MX2, and ISG15 antibodies; Mac-TREM2 with anti-C1q-FITC conjugated antibody and pDCs with anti-HLA-DR and anti-CD123 antibodies to validate the in vivo expression of these markers in SARS CoV-2 infected lung tissue (Table S2). SARS CoV-2 nucleocapsid antibody was used to detect SARS CoV-2 and ACE-2 expression was confirmed using human anti-ACE2 antibody. DAPI was used for nuclear staining. Images were captured on Ziess LSM-800 confocal microscope using Olympus cellSens Entry Imaging Software Version 1.18 and ZEN Imaging software version 2.1 (blue edition).

Quantification of various cellular phenotypes in FFPE sections was done using the ‘Indica labs- HighPlex FL v4.1.3’ algorithm in HALO v3.3 (Indica labs). Briefly, for each staining set, we started with inputting the number and name of dyes as well as the number of phenotypes required for output. Thereafter, nuclear parameters including size, roundness, segmentation, nuclear dyes, minimum and maximum intensity were entered. Similarly, cellular parameters, namely cytoplasm radius, membrane segmentation, cell size were set. For entraining the algorithm, the minimum and maximum fluorescence intensities were selected for each individual dye used in the staining. Finally, the required phenotypes together with their respective criteria (channels and filters) were added. The entire slide scanned using Axio Scan Z1 (Zeiss) were then analyzed using the trained algorithm to obtain the output as per the above selected phenotypes. The number of cells for each phenotype per unit area were then plotted using GraphPad Prism v8.4.3 (EDF5).

### Statistics and reproducibility

Graphs were prepared and statistical comparisons were applied using GraphPad Prism v8.4.3. Statistical comparisons were performed as outlined in respective methods. One-way repeated measure ANOVA with Geisser Greenhouse correction for sphericity and Tukey post hoc correction for multiple testing (GraphPad Prism v8.4.3) was applied for statistical comparison of population clusters across timepoints as described in the figure legends. For correlation analysis, Spearman’s rank tests were applied. Statistical differences between groups were reported to be significant when the *P*-value was ≤0.05. Data are presented as mean ± standard error of the mean (SEM). scRNAseq was performed on longitudinal BAL cells from six young macaques across all timepoints and no samples were excluded. For validation of scRNAseq dataset, immuno-histochemistry was performed on Naive (four randomly selected lung lobes from four macaques) and SARS CoV-2 infected Rhesus macaque lungs at Day 3 (four randomly selected lung lobes from two macaques) and Day 14–17 (four randomly selected lung lobes from four macaques) post-infection. three random fields were acquired for each section analyzed.

### Reporting summary

Further information on research design is available in the Nature Research Reporting Summary linked to this article.

## Supplementary information

Supplementary Information

Peer Review File

Description of Additional Supplementary Information

Supplementary Dataset 1

Supplementary Dataset 2

Supplementary Dataset 3

Reporting Summary

## Data Availability

Source data are provided with this paper. All data supporting the findings of this study are available within this manuscript and its Supplementary Information. Any additional data can be requested from the corresponding authors upon reasonable request. The scRNAseq raw data generated in this study have been deposited to the Gene Expression Omnibus-GEO (NCBI) under accession number GSE190659 (https://www.ncbi.nlm.nih.gov/geo/query/acc.cgi?acc=GSE190659). The IHC data generated in this study have been deposited in the Figshare database and can be accessed at this link 10.6084/m9.figshare.17197925.
