# Supplementary material for: Myeloid cell interferon responses correlate with clearance of SARS-CoV-2
Source: Nat Commun. 2022 Feb 3;13:679. doi: 10.1038/s41467-022-28315-7 (PMC8814034; doi:10.1038/s41467-022-28315-7)
Supplement: Supplementary file 1 — Supplementary Information [file 41467_2022_28315_MOESM1_ESM.pdf]

Myeloid cell interferon responses correlate with clearance of SARS-CoV-2

Dhiraj K. Singh*[1], Ekaterina Aladyeva*[2], Shibali Das*[3], Bindu Singh[1], Ekaterina Esaulova[2], Amanda Swain[2], Mushtaq Ahmed[3], Journey Cole[1], Chivonne Moodley[1,4], Smriti Mehra[1], Larry S. Schlesinger[1], Maxim N. Artyomov[2]**, Shabaana A. Khader[3]** and Deepak Kaushal[1]**

[1]Southwest National Primate Research Center, Texas Biomedical Research Institute, San Antonio, TX 78245.
[2]Department of Medicine, Washington University in St. Louis, St. Louis, MO 63110.
[3]Department of Molecular Microbiology, Washington University in St. Louis, St. Louis, MO 63110.
[4]Tulane National Primate Research Center, Tulane University School of Medicine, Covington, LA 70433.

* These authors contributed equally
** These authors jointly supervised this work
Corresponding authors: Deepak Kaushal, Southwest National Primate Research Center, Texas Biomedical Research Institute, San Antonio, TX 78245, dkaushal@txbiomed.org; Shabaana A. Khader, Department of Molecular Microbiology, Washington University in St. Louis, St. Louis, MO 63110, sakhader@wustl.edu; and Maxim N. Artyomov, PhD, Department of Pathology and Immunology, Washington University School of Medicine, St. Louis, MO 63110, martyomov@wustl.edu.

Supplementary Information

Supplementary Figure 1

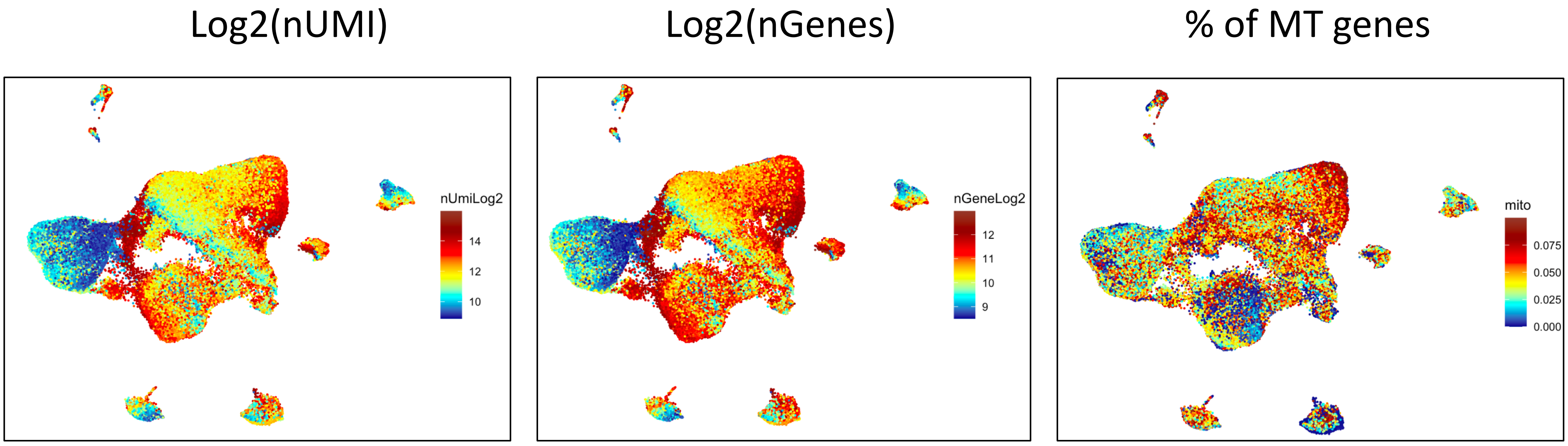

Supplementary Figure 1. UMAP plots representing QC parameters for analysis. (A) nUMI, (B) nGenes and (C) fraction of mitochondrial genes.

Supplementary Figure 2

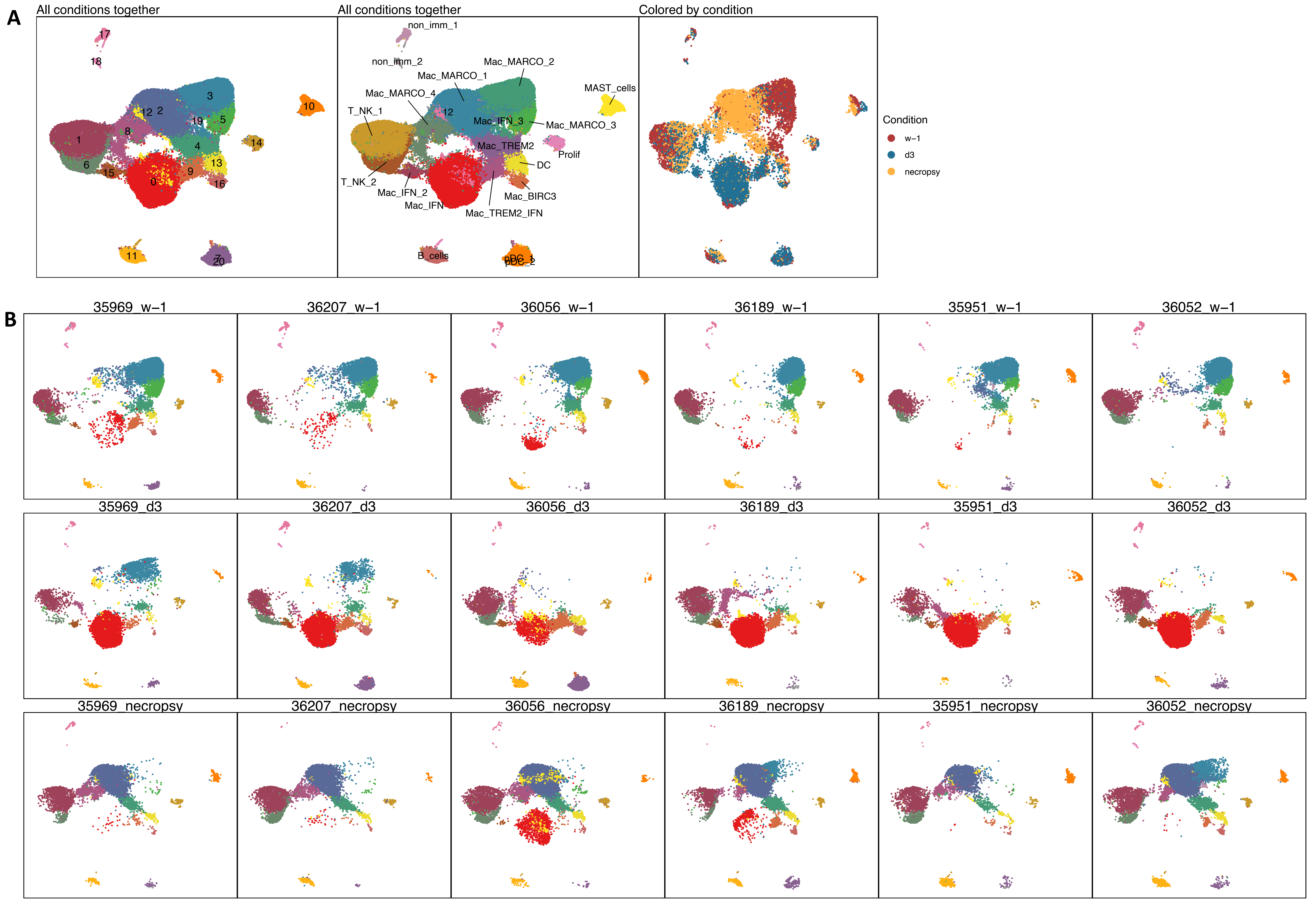

Supplementary Figure 2. (A) UMAP plots depicting all clusters (left), cluster annotations (middle), distribution as per condition (right) and (B) distribution per sample.

Supplementary Figure 3

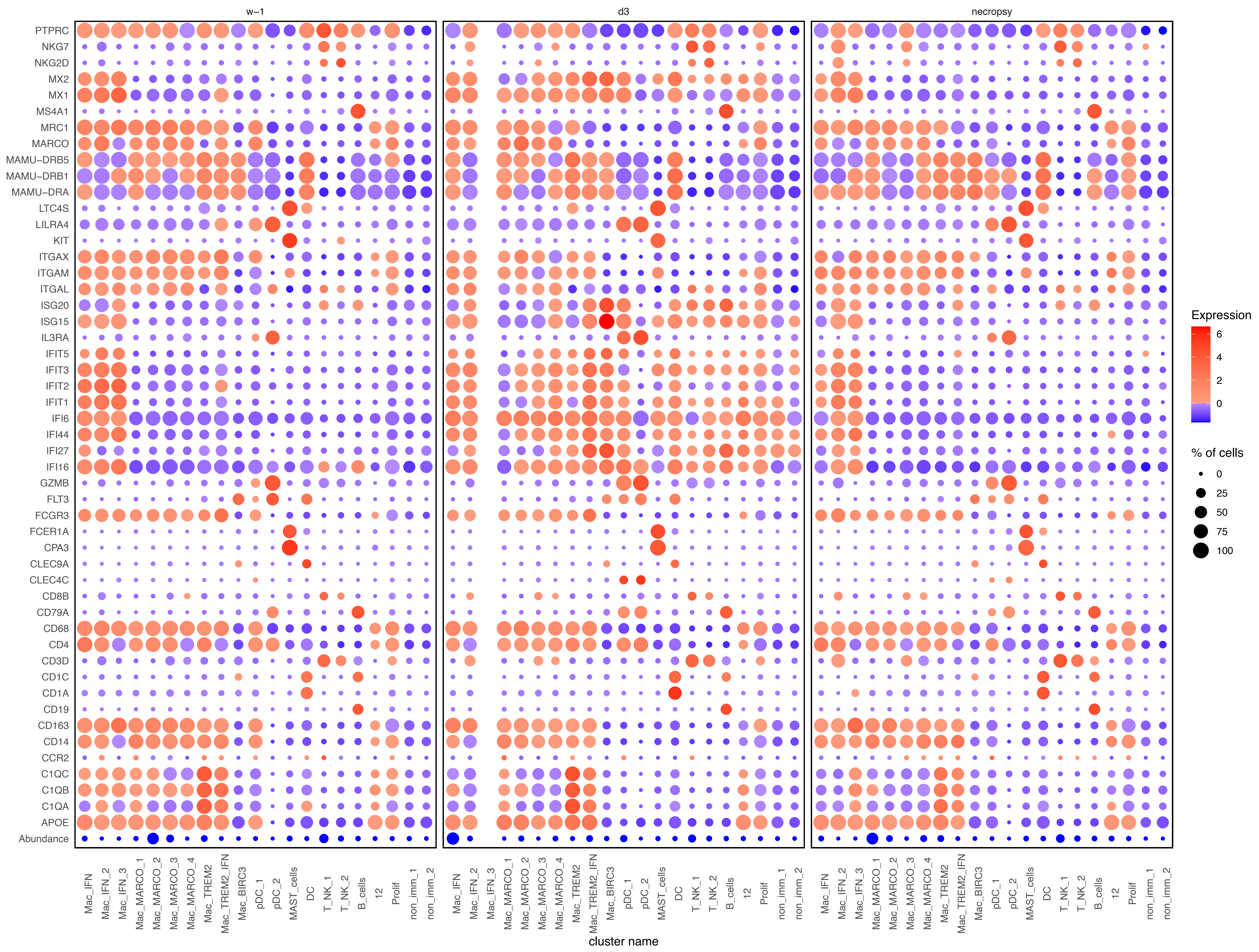

Supplementary Figure 3. Bubble plot showing the fractional abundance of identified clusters (bottom row), fold change of genes and the fraction of cells expressing the gene of interest in different phases of COVID-19.

Supplementary Figure 4

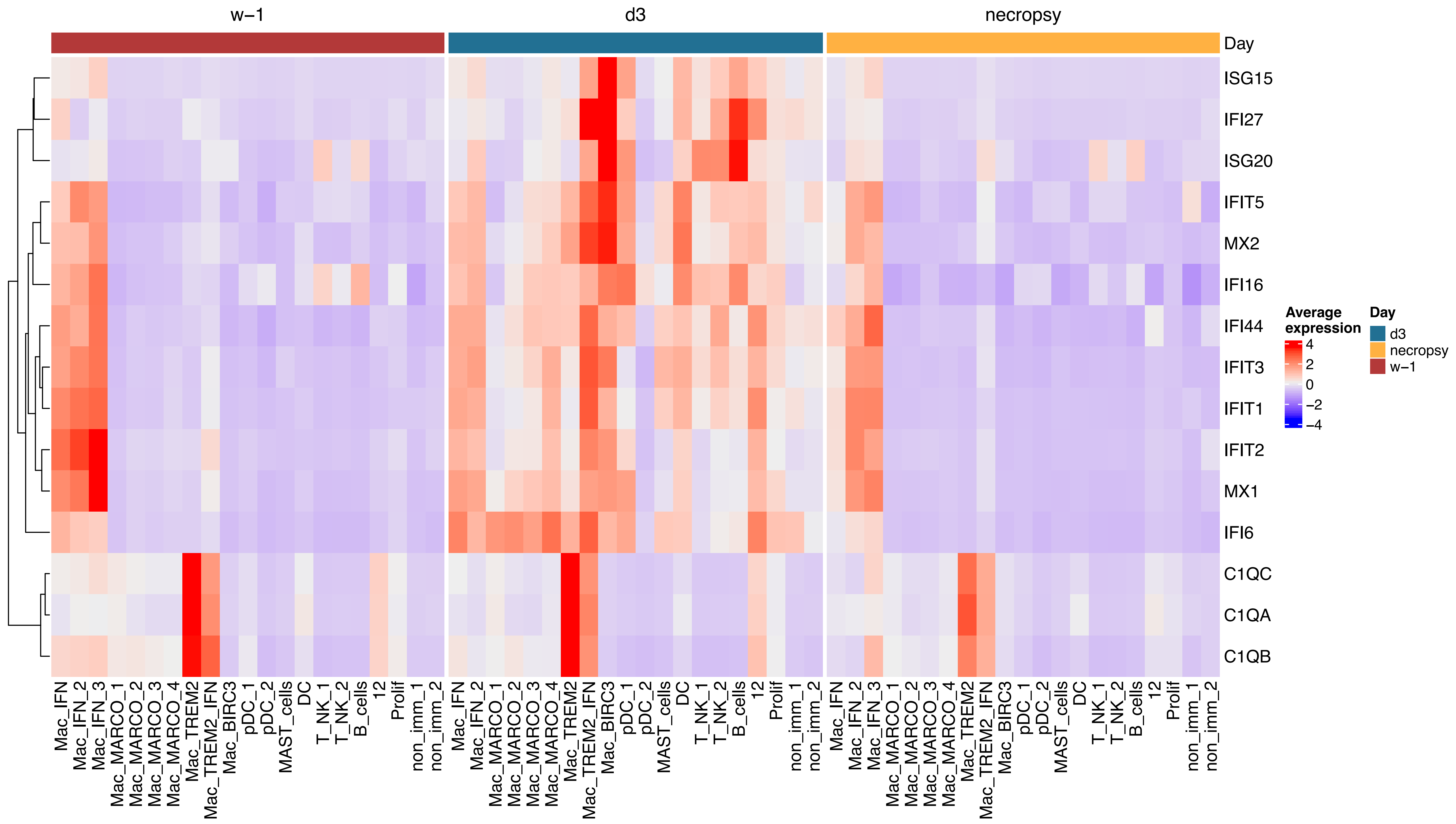

Supplementary Figure 4. Heatmap representing differential expression of genes of interest in identified clusters in different phases of COVID-19.

Supplementary Figure 5

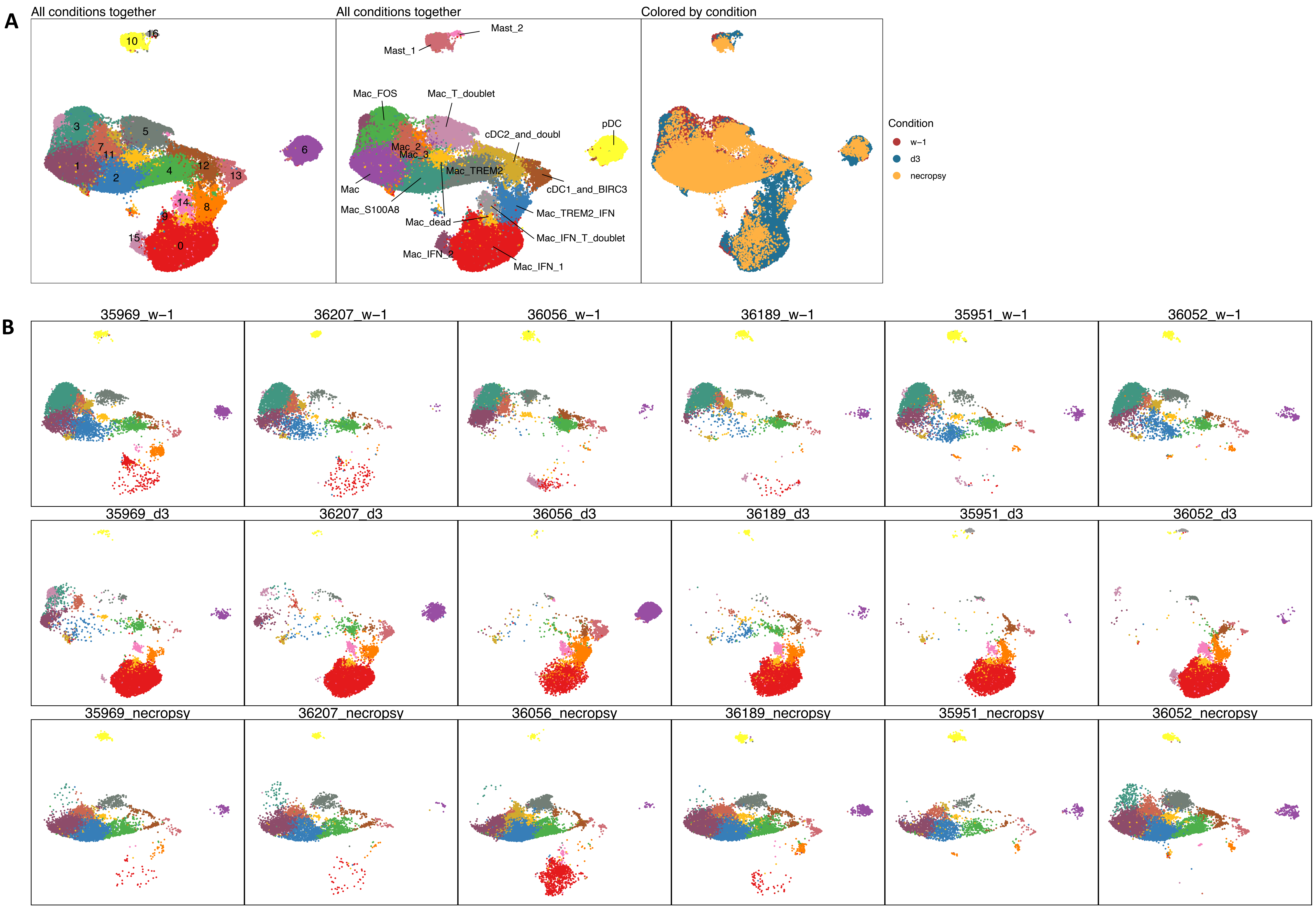

Supplementary Figure 5. (A) UMAP plots depicting all myeloid clusters (left), cluster annotations (middle), distribution as per condition (right) and (B) distribution per sample.

Supplementary Figure 6

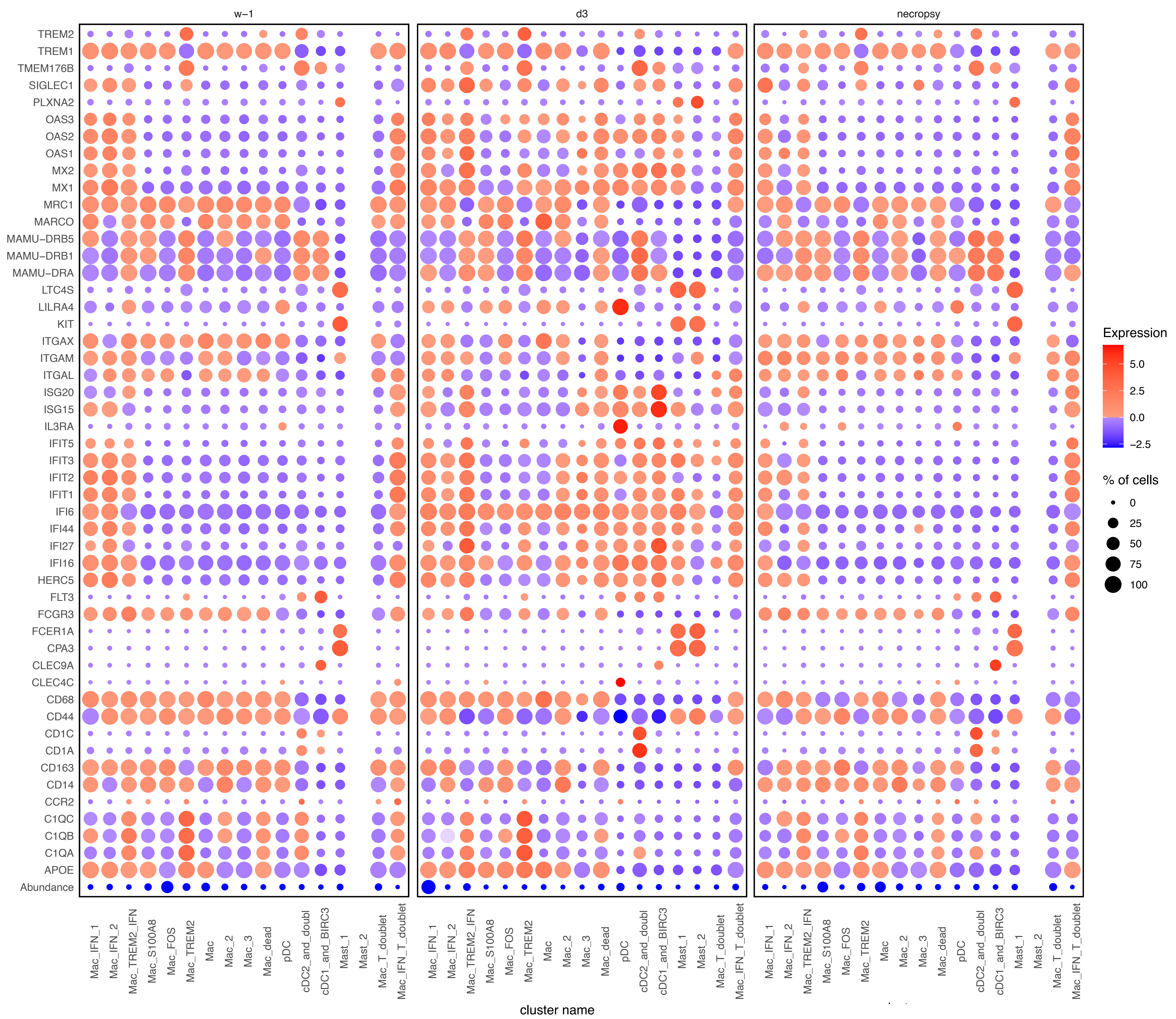

Supplementary Figure 6. Bubble plot showing the fractional abundance of identified myeloid clusters (bottom row), fold change of genes and the fraction of cells expressing the gene of interest in different phases of COVID-19.

Supplementary Figure 7

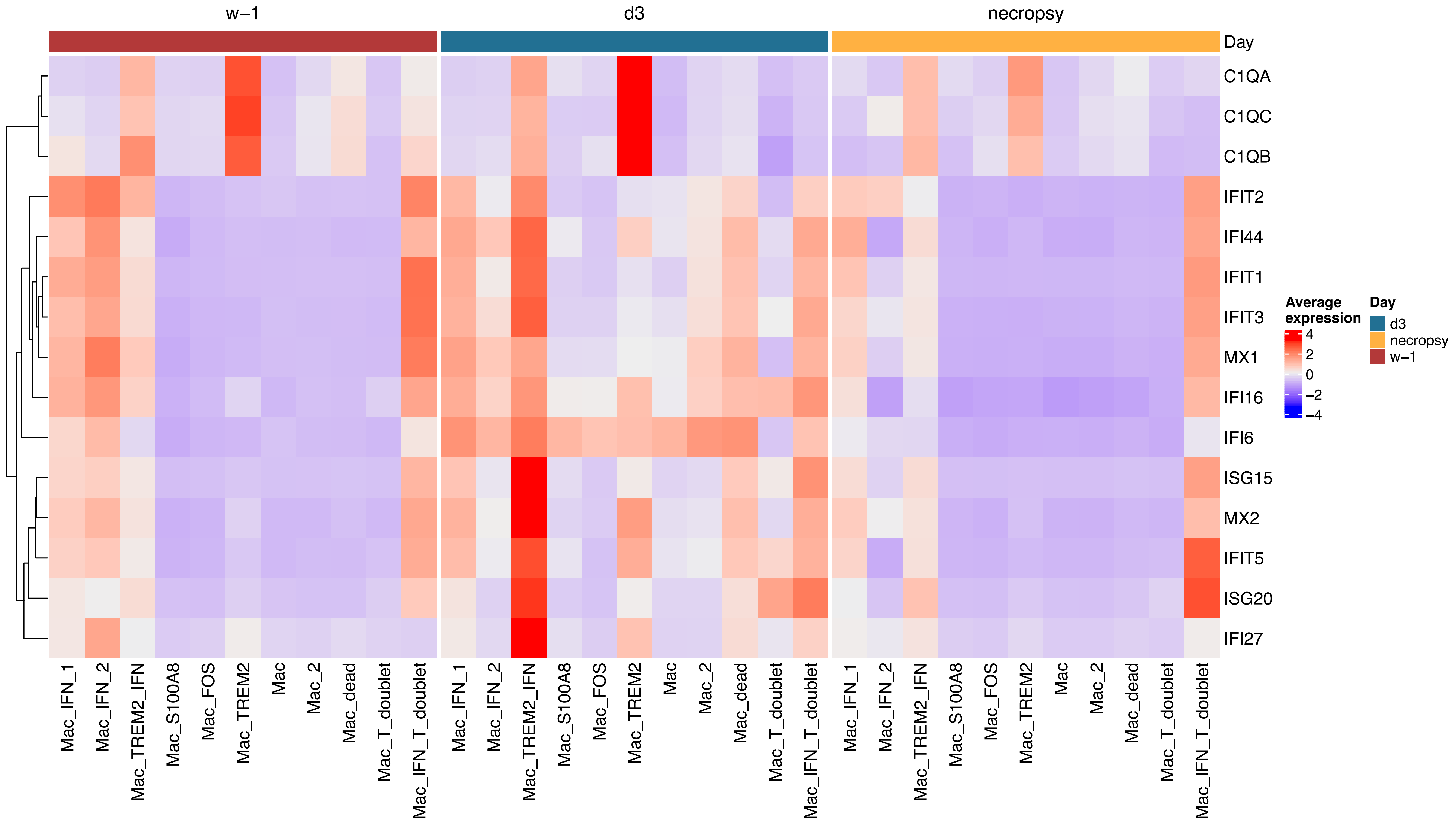

Supplementary Figure 7. Heatmap representing differential expression of genes of interest in identified myeloid clusters in different phases of COVID-19.

Supplementary Figure 8

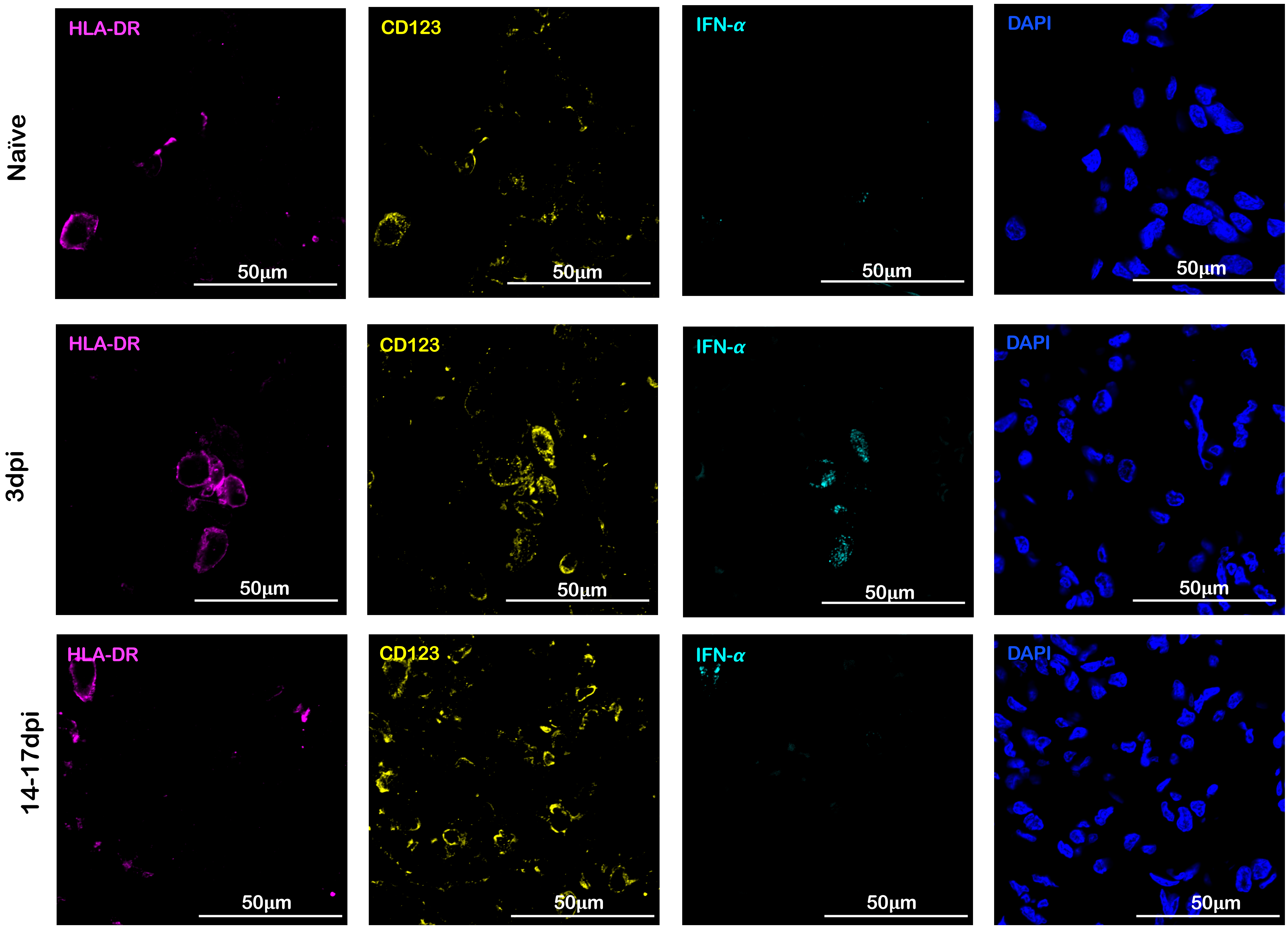

Supplementary Figure 8. Single channel images depicting pDCs marked with HLA-DR (magenta) and CD123 (yellow) showing expression of IFN-α (turquoise) in Naïve lungs as well as SARS CoV-2 infected lungs at Day 3 and Day 14 post-infection at 63x magnification. Nuclei stained with DAPI are shown in blue.

Supplementary Figure 9

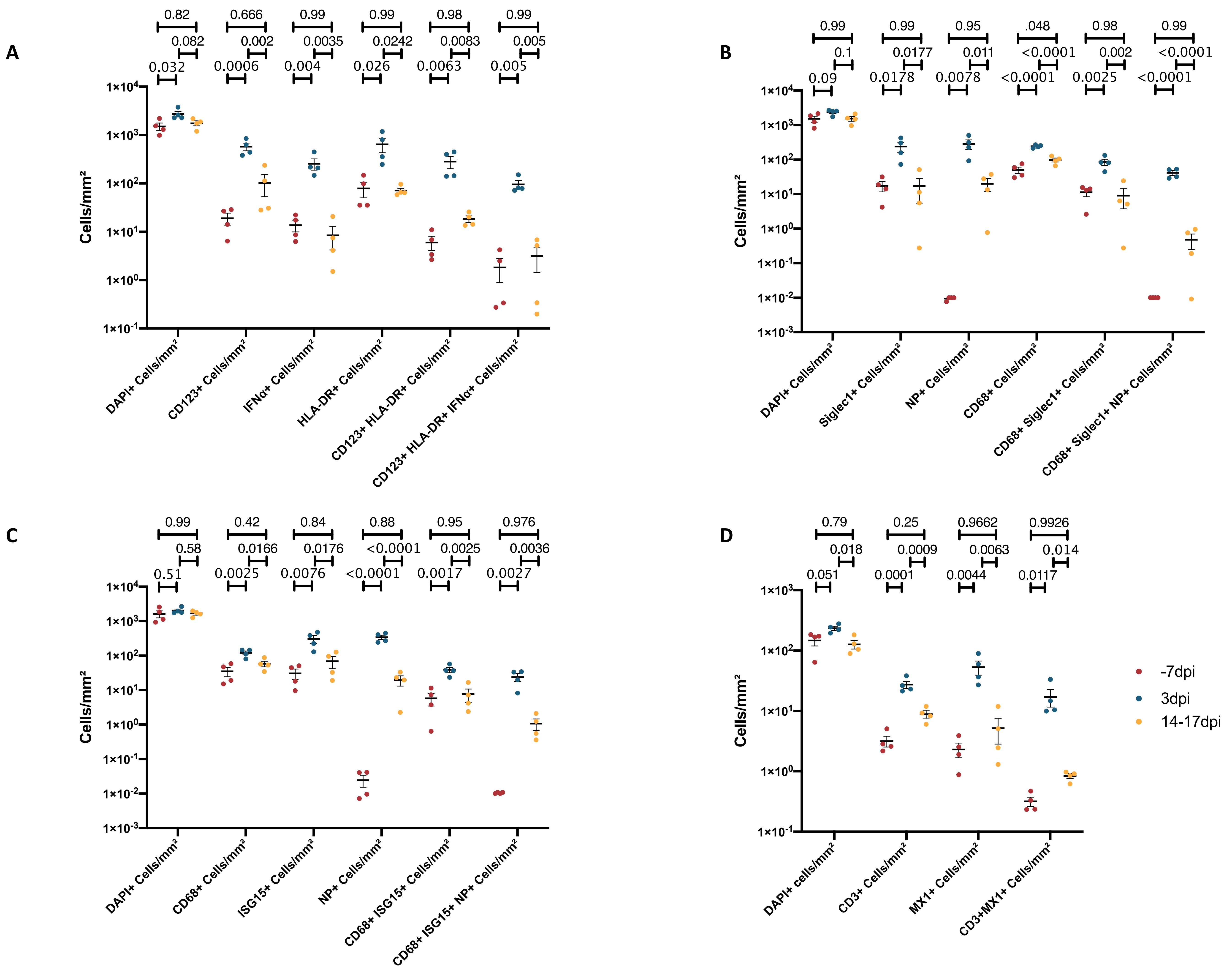

Supplementary Figure 9. Quantification of immunofluorescence confocal images showing (A) pDC expressing IFN-α, (B) SIGLEC1+ and NP+ Macrophages, (C) ISG15+ and NP+ Macrophages, (D) MX1+ T cells. Quantification was performed on Naïve (4 randomly selected lung lobes from 4 macaques) and SARS CoV-2 infected Rhesus macaque lungs at Day 3 (4 randomly selected lung lobes from 2 macaques) and Day 14-17 (4 randomly selected lung lobes from 4 macaques) post-infection. Data are presented as mean ± standard error of the mean (SEM). Source data are provided as a Source Data file.

Supplementary Figure 10

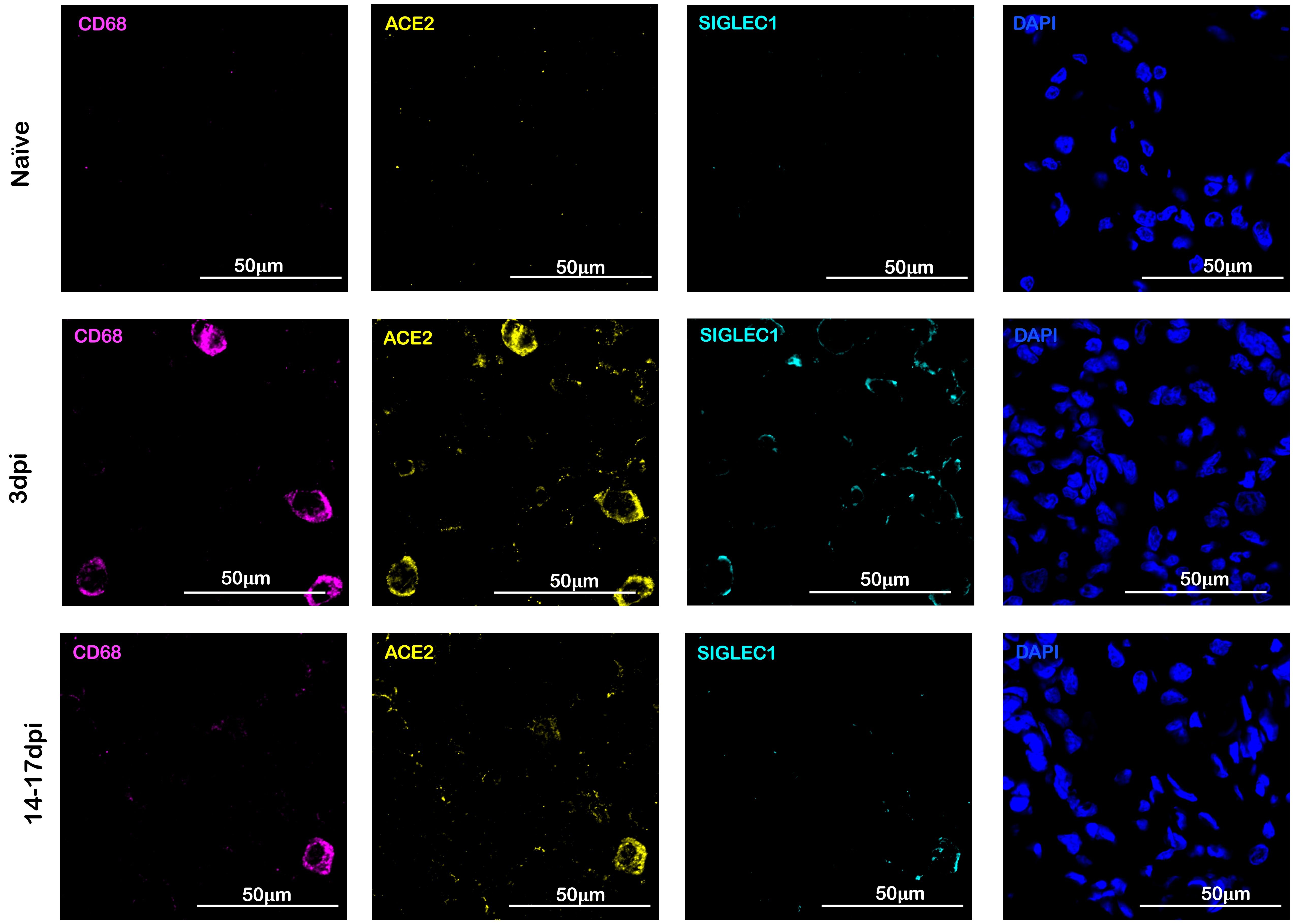

Supplementary Figure 10. Single channel images validating the expression of ACE2 receptor (yellow) on CD68 (magenta) and SIGLEC1 (turquoise) positive macrophages in Naïve and SARS CoV-2 infected lungs (3dpi and 14-17dpi).

Supplementary Figure 11

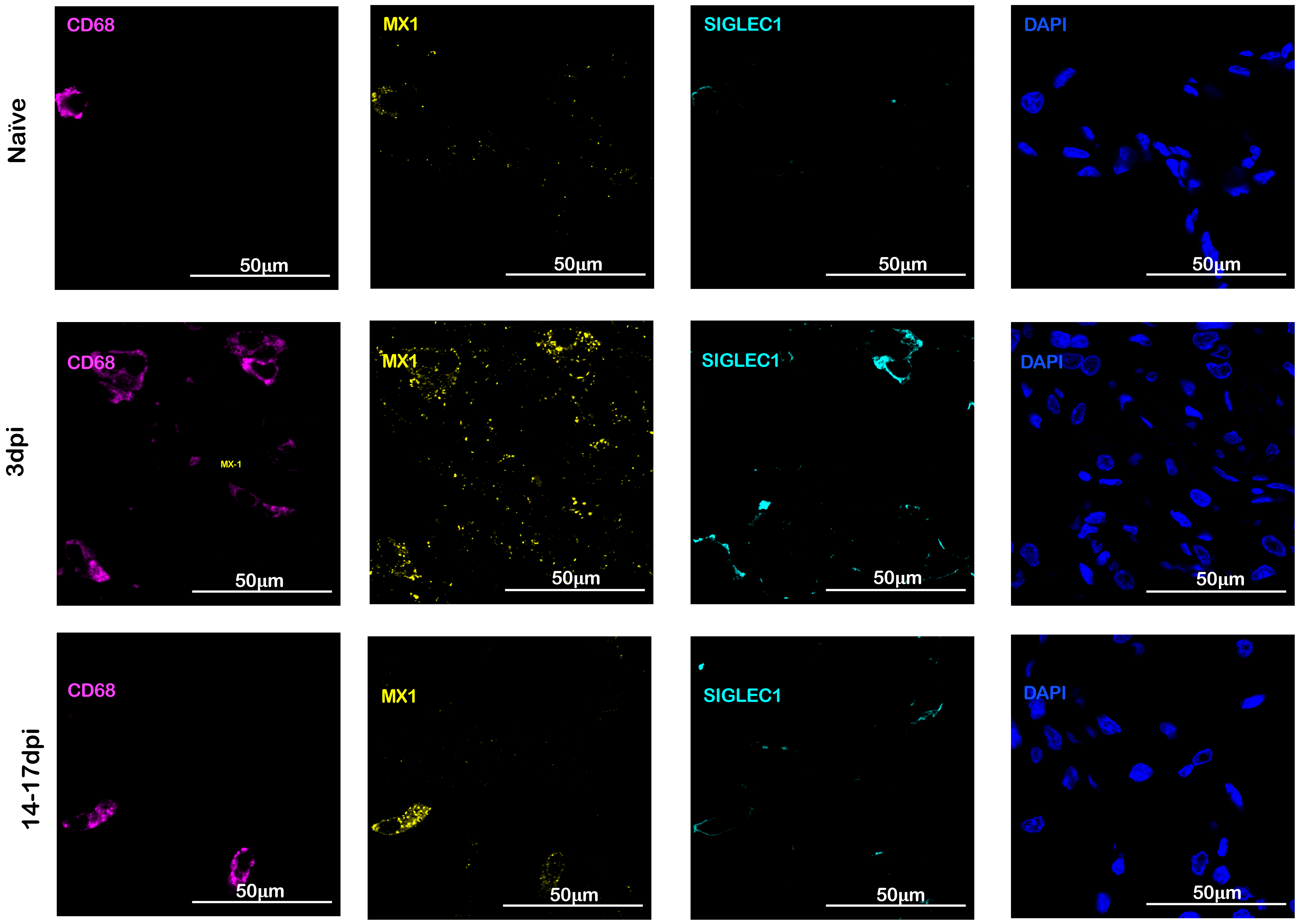

Supplementary Figure 11. Images showing separate channels for expression of Mac-IFN signature marker MX1 (yellow) by CD68 (magenta) and SIGLEC1 (turquoise) positive macrophages in Naïve as well as 3dpi and 14-17dpi SARS CoV-2 infected macaque lungs.

Supplementary Figure 12

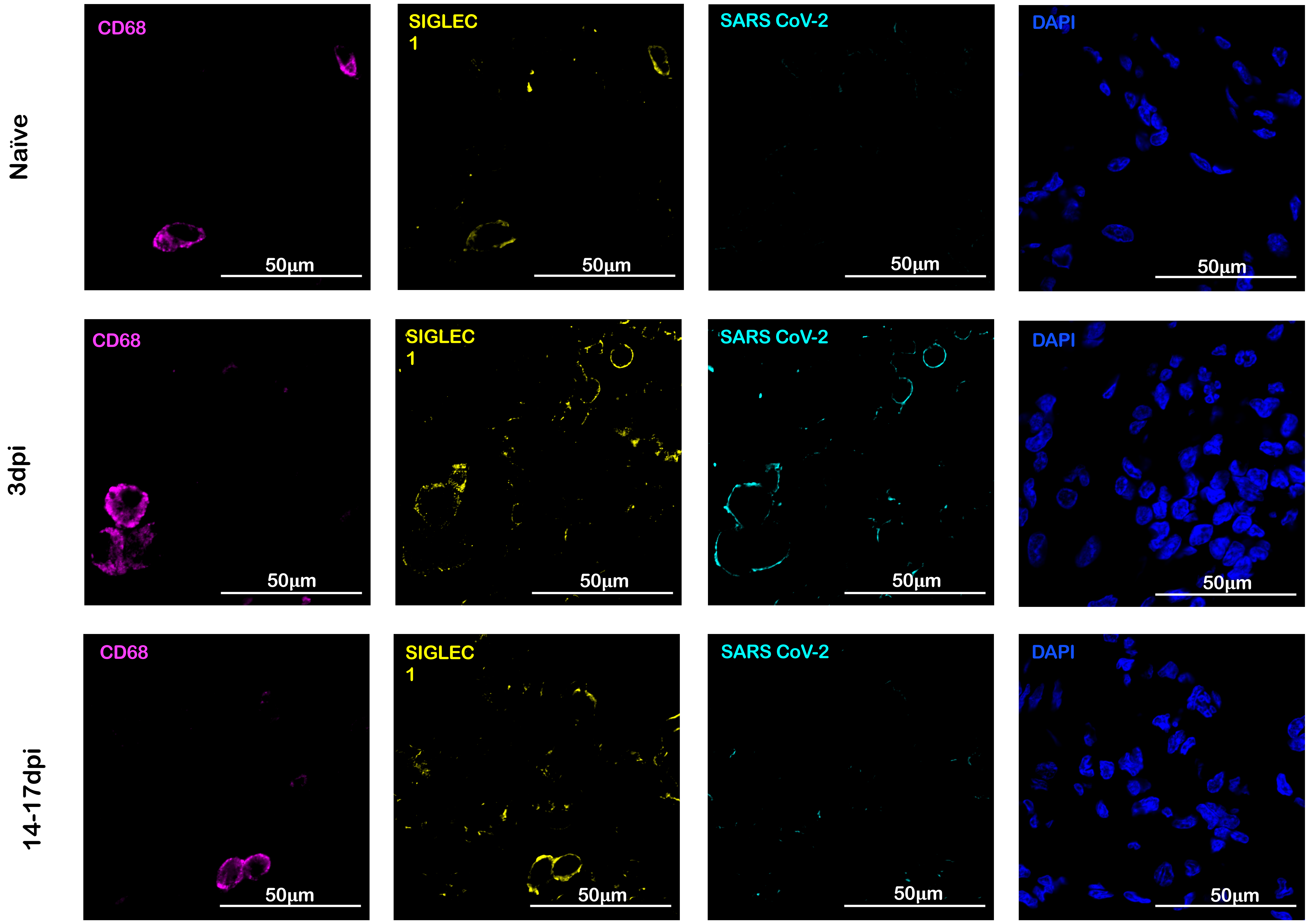

Supplementary Figure 12. Single color images from Naïve and SARS CoV-2 infected lungs (3dpi and 14-17dpi) depicting SARS CoV-2 (turquoise) is harbored by CD68 (magenta) and SIGLEC1 (yellow) positive macrophages.

Supplementary Figure 13

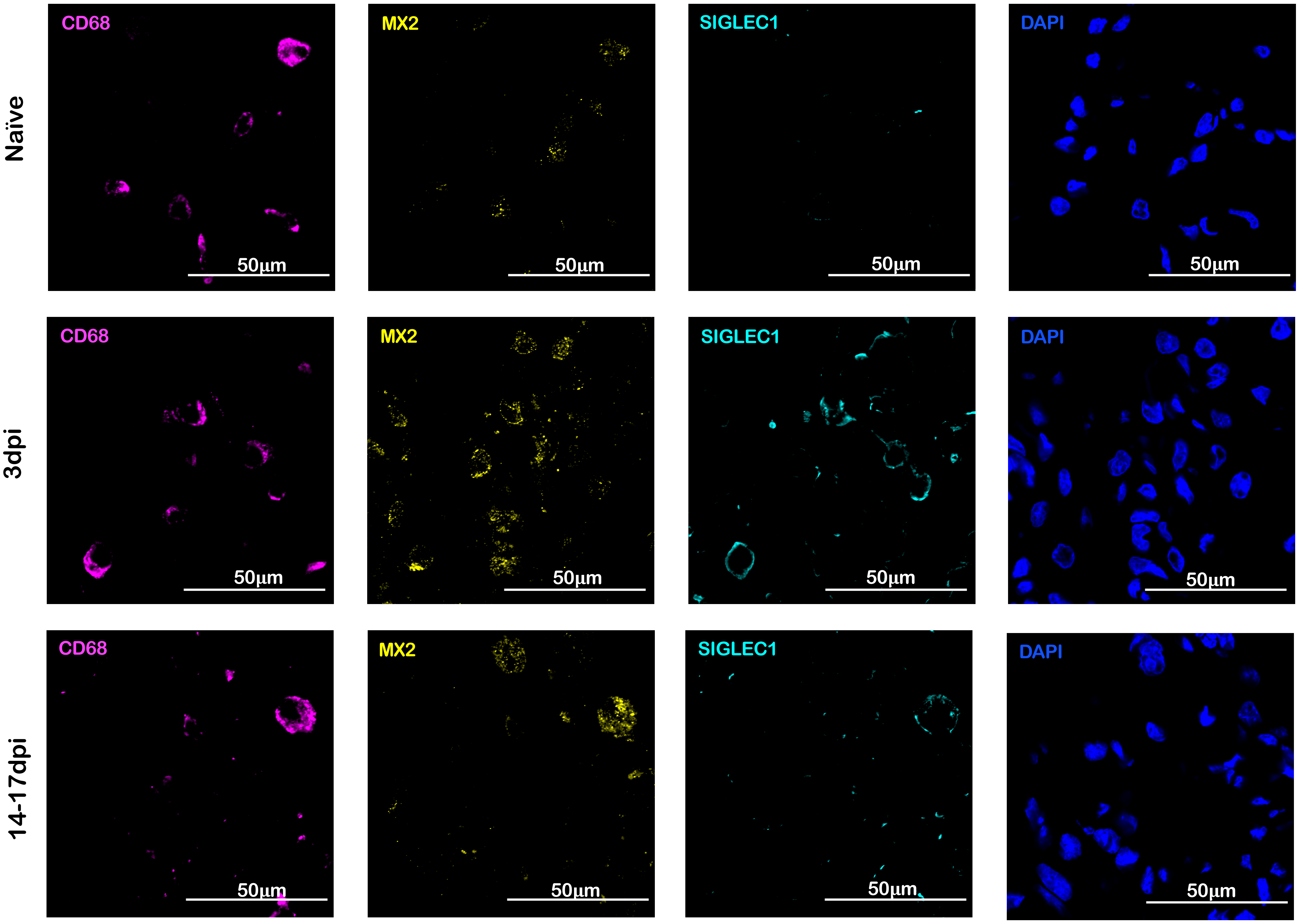

Supplementary Figure 13. Shown are the images for single channels for CD68 (magenta) and SIGLEC1 (turquoise) positive macrophages expressing MX2 (yellow), one of the Mac-IFN signature markers. Images from Naïve as well as SARS CoV-2 infected lungs at Day 3 and Day 14 post-infection were taken to see the differential expression.

Supplementary Figure 14

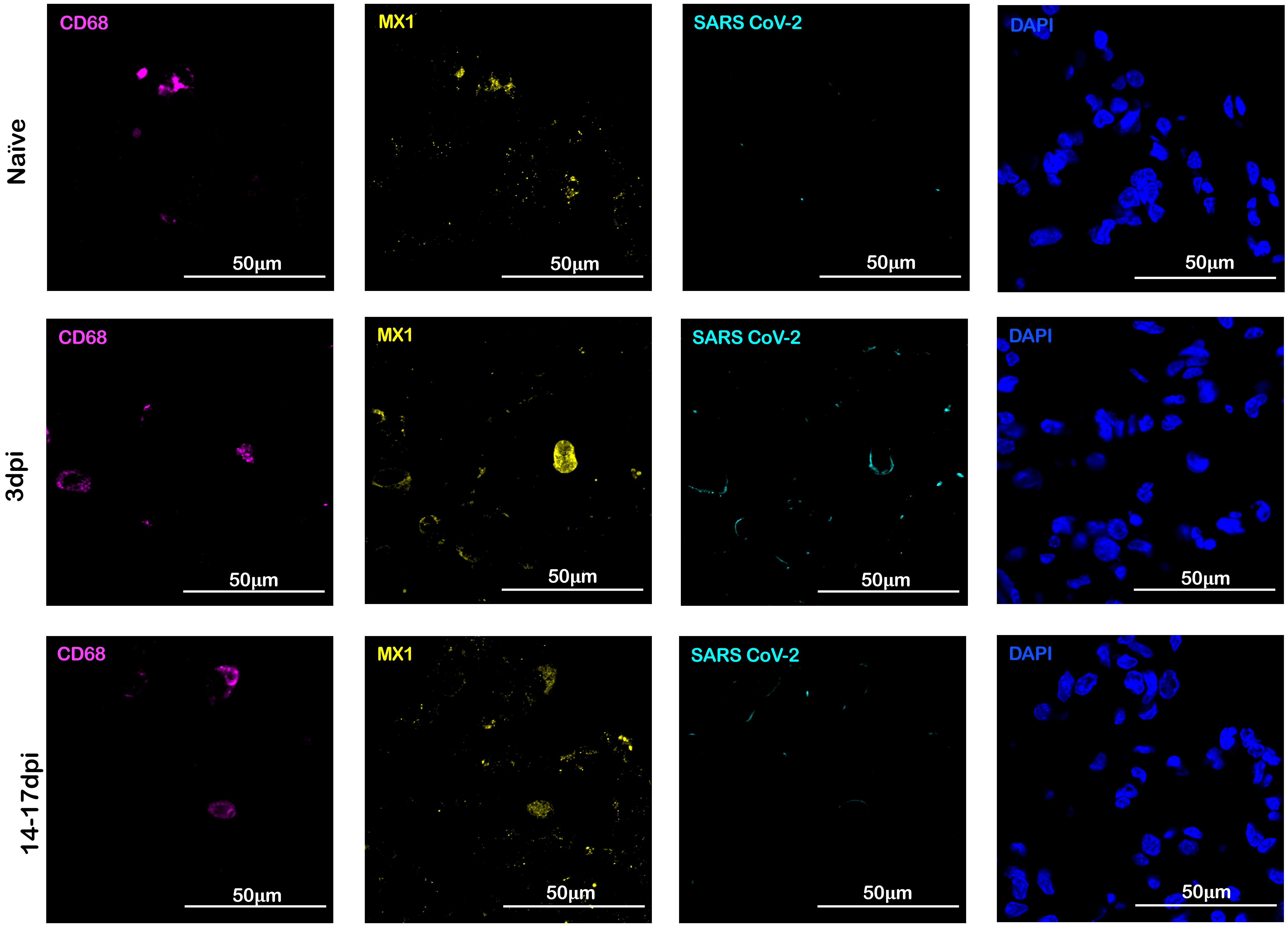

Supplementary Figure 14. Shown are the single-channel images from Naïve and SARS CoV-2 infected lung sections (3dpi and 14-17dpi) depicting CD68 (magenta) and MX1 (yellow) positive macrophages harbors SARS CoV-2 (turquoise).

Supplementary Figure 15

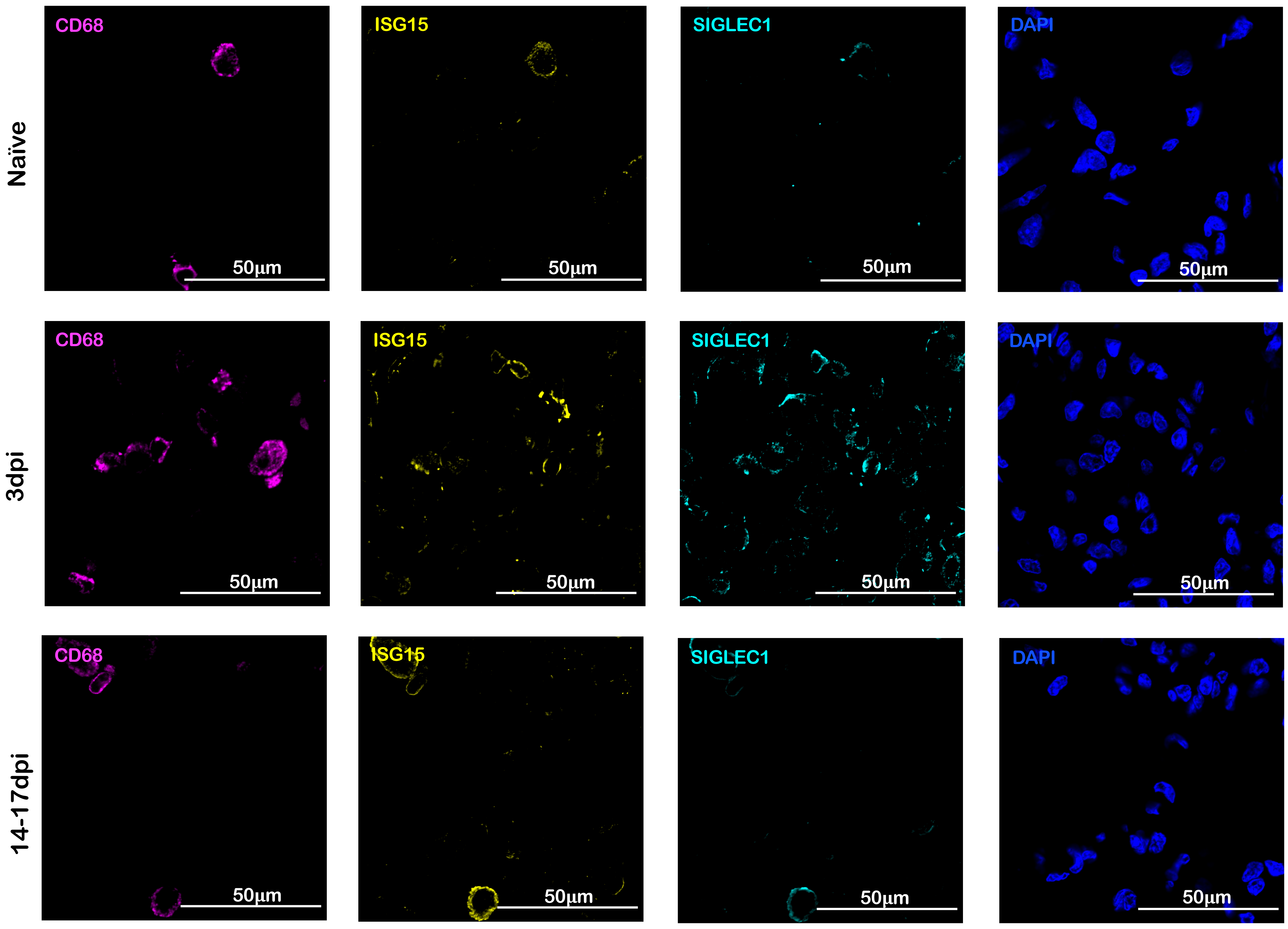

Supplementary Figure 15. Single color images from the Naïve and SARS CoV-2 infected lungs sections (at 3dpi and 14-17dpi) showing CD68 (magenta) and SIGLEC1 (turquoise) positive macrophages expressing ISG15 (yellow), another Mac-IFN signature marker.

Supplementary Figure 16

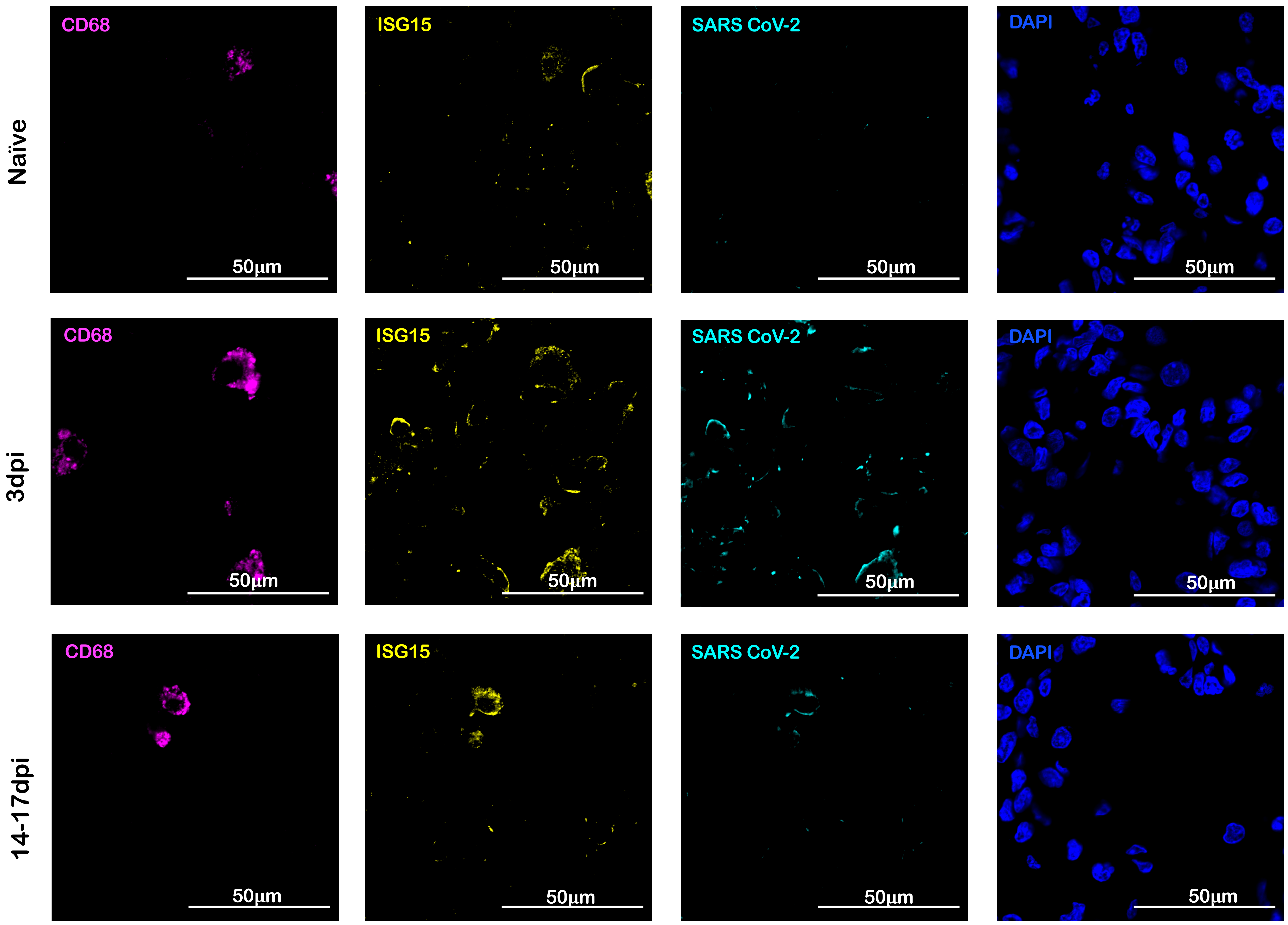

Supplementary Figure 16. Single color images depicting ISG15 (yellow) expressing macrophages (magenta) harbor SARS CoV-2 (turquoise). Images shown are from the Naïve and SARS CoV-2 infected lung sections both at 3dpi and 14-17dpi.

Supplementary Figure 17

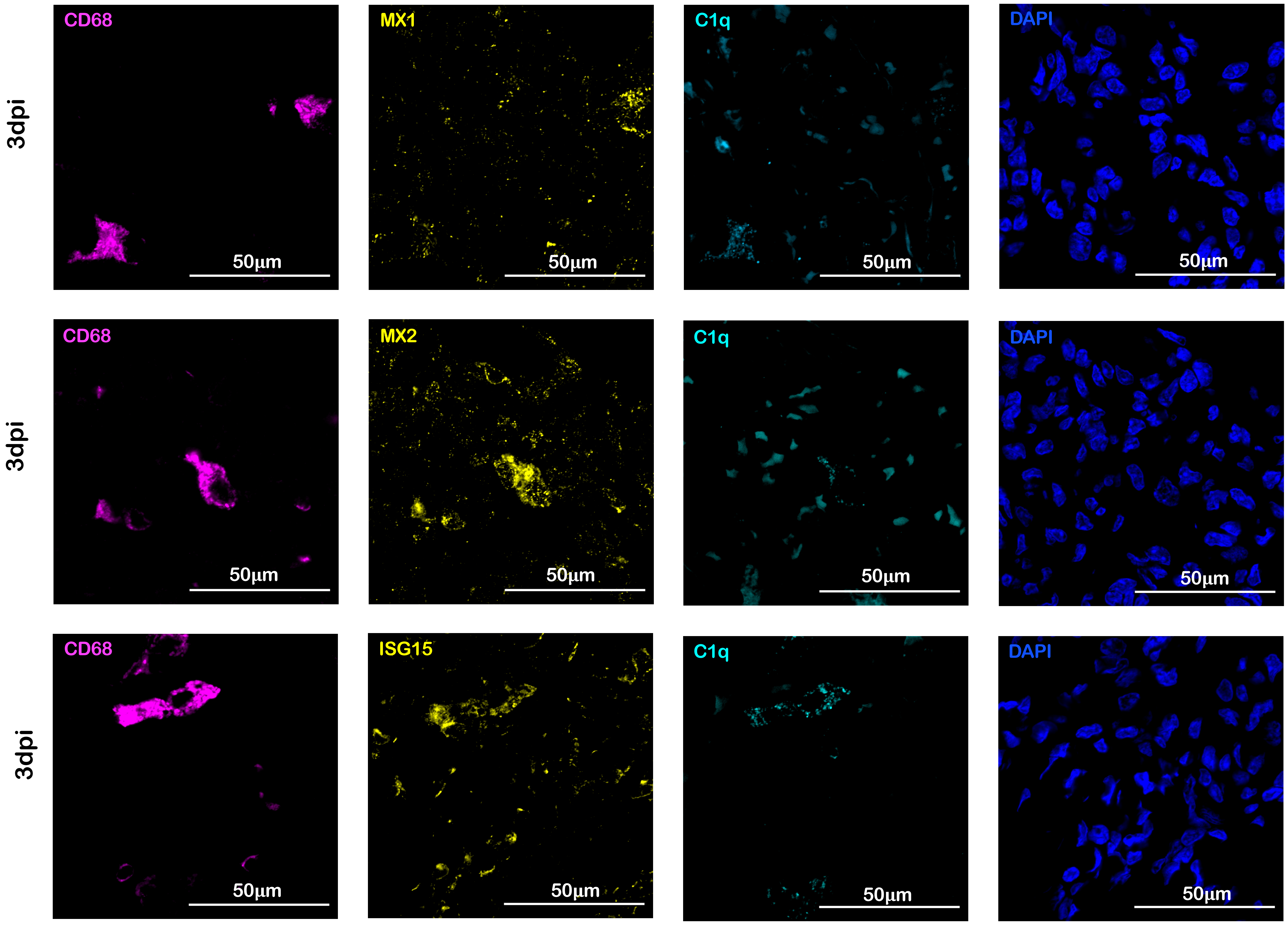

Supplementary Figure 17. Single channel images from lungs of SARS CoV-2 infected macaques at 3dpi showing the expression of Mac-IFN signature markers: MX1, MX2 and ISG15 (yellow) and TREM marker: C1q (turquoise) in CD68 positive macrophages (magenta).

Supplementary Figure 18

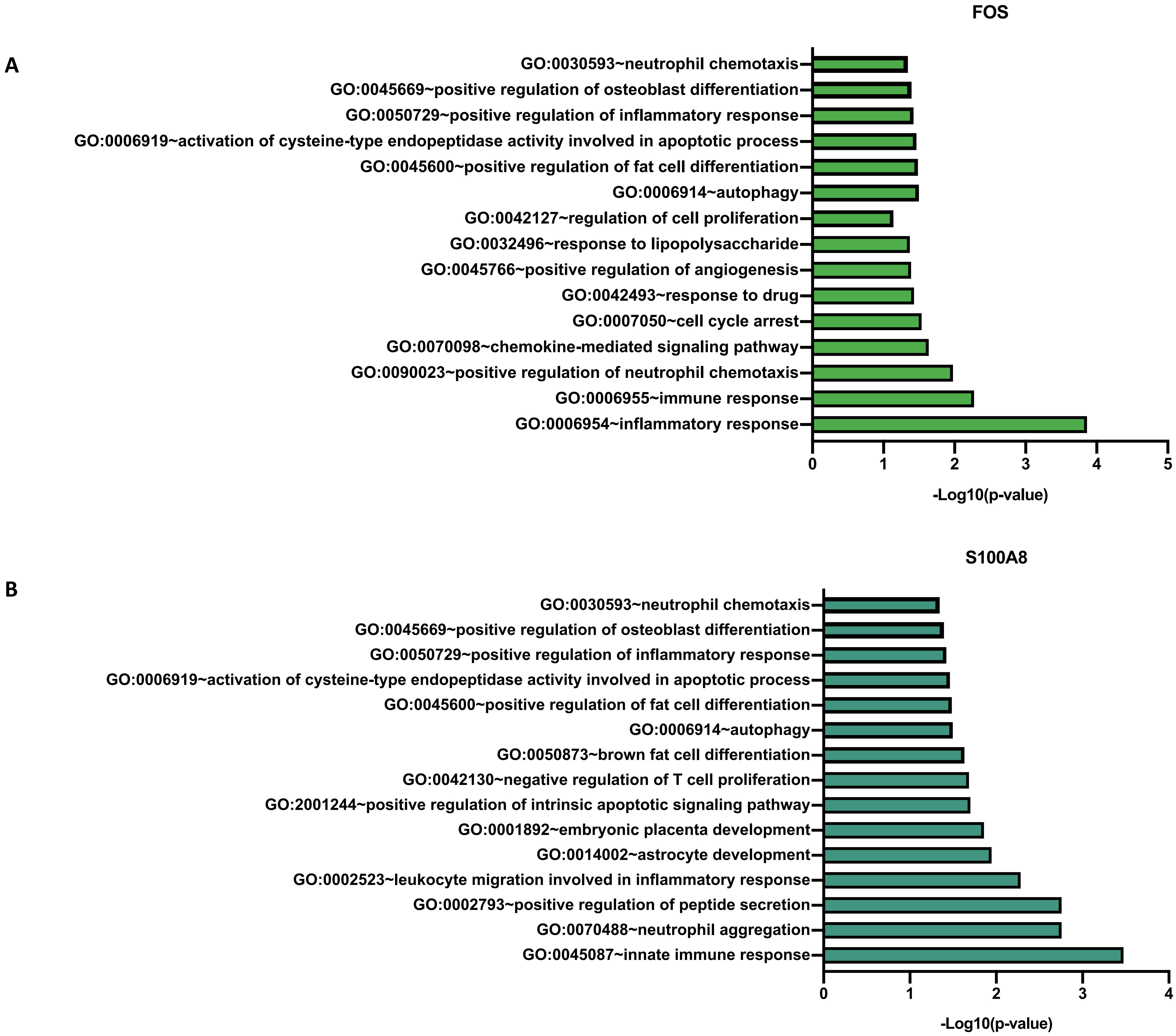

Supplementary Figure 18. GSEA enrichment set in (A) Mac_FOS and (B) Mac_S100A8 cluster.

Supplementary Figure 19

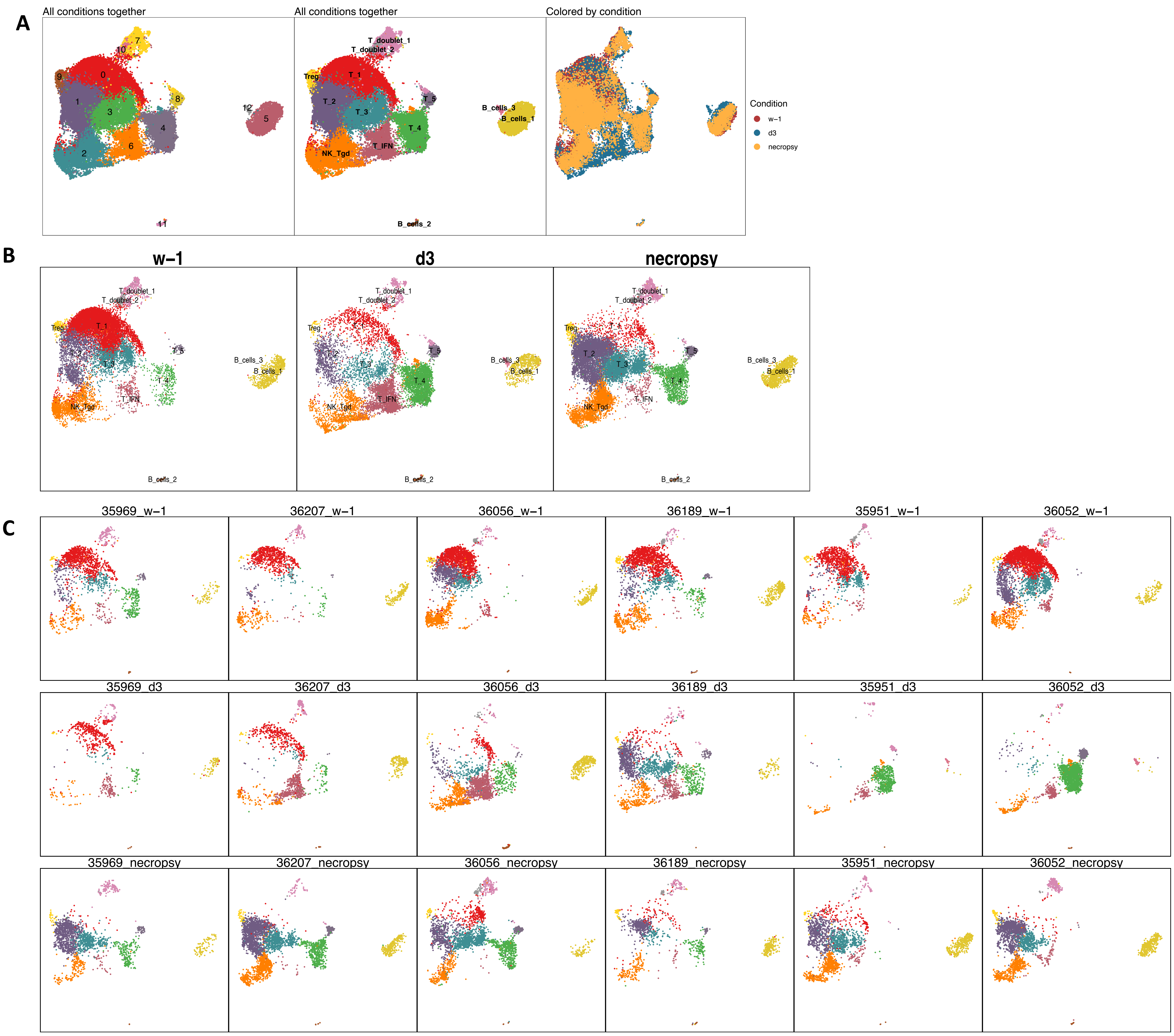

Supplementary Figure 19. (A) UMAP plots depicting all lymphoid clusters (left), cluster annotations (middle), distribution as per condition (right), (B) respective timepoints. and (C) distribution per sample.

Supplementary Figure 20

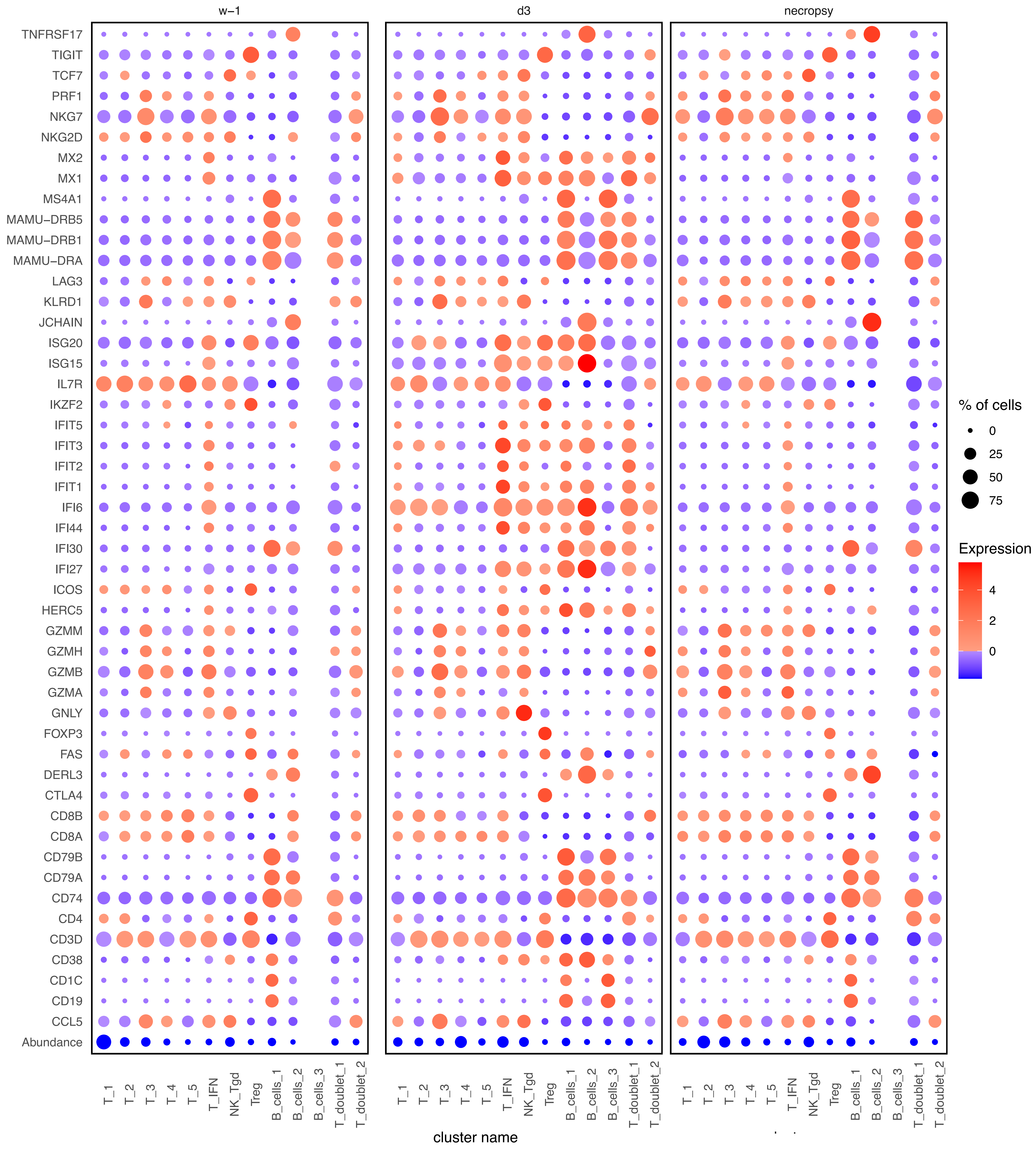

Supplementary Figure 20. Bubble plot showing the fractional abundance of identified lymphoid clusters (bottom row), fold change of genes and the fraction of cells expressing the gene of interest in different phases of COVID-19.

Supplementary Figure 21

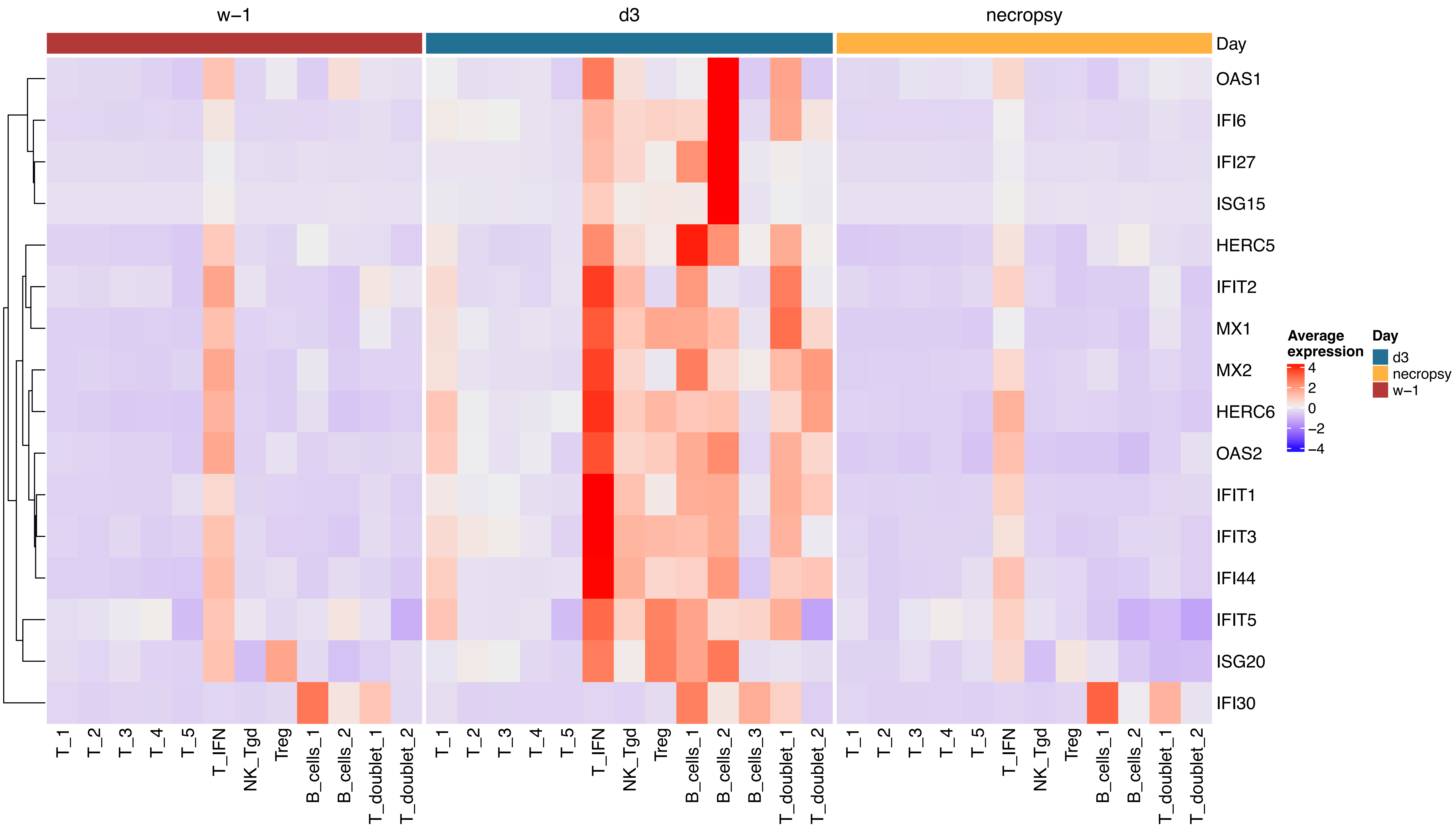

Supplementary Figure 21. Heatmap representing differential expression of genes of interest in identified lymphoid clusters in different phases of COVID-19.

Supplementary Figure 22

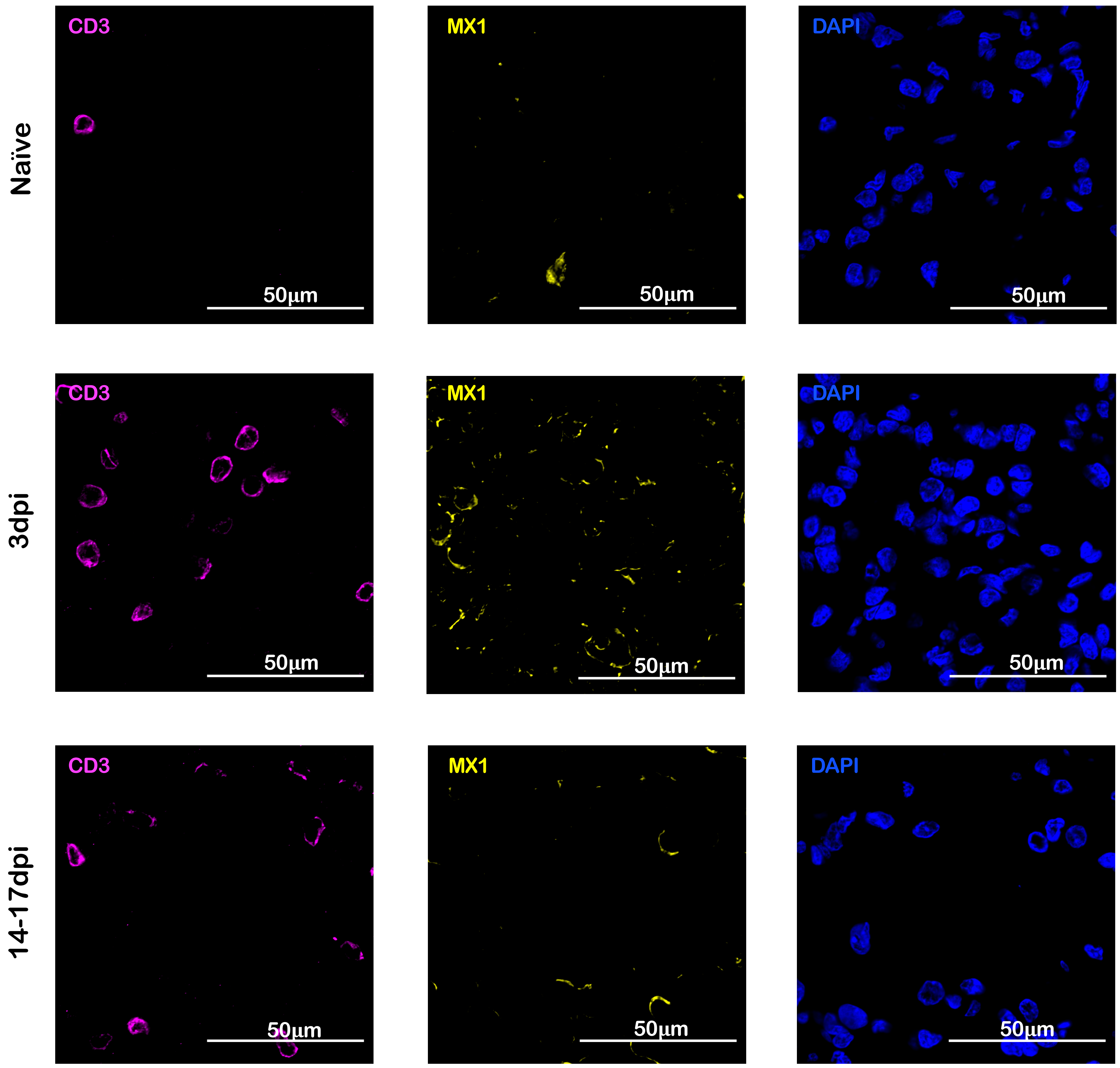

Supplementary Figure 22. Images showing single channels from CD3 (magenta) and MX1 (yellow) stained lungs sections from Naïve and SARS CoV-2 infected macaques at Day 3 and Day 14 post-infection.

Supplementary Figure 23

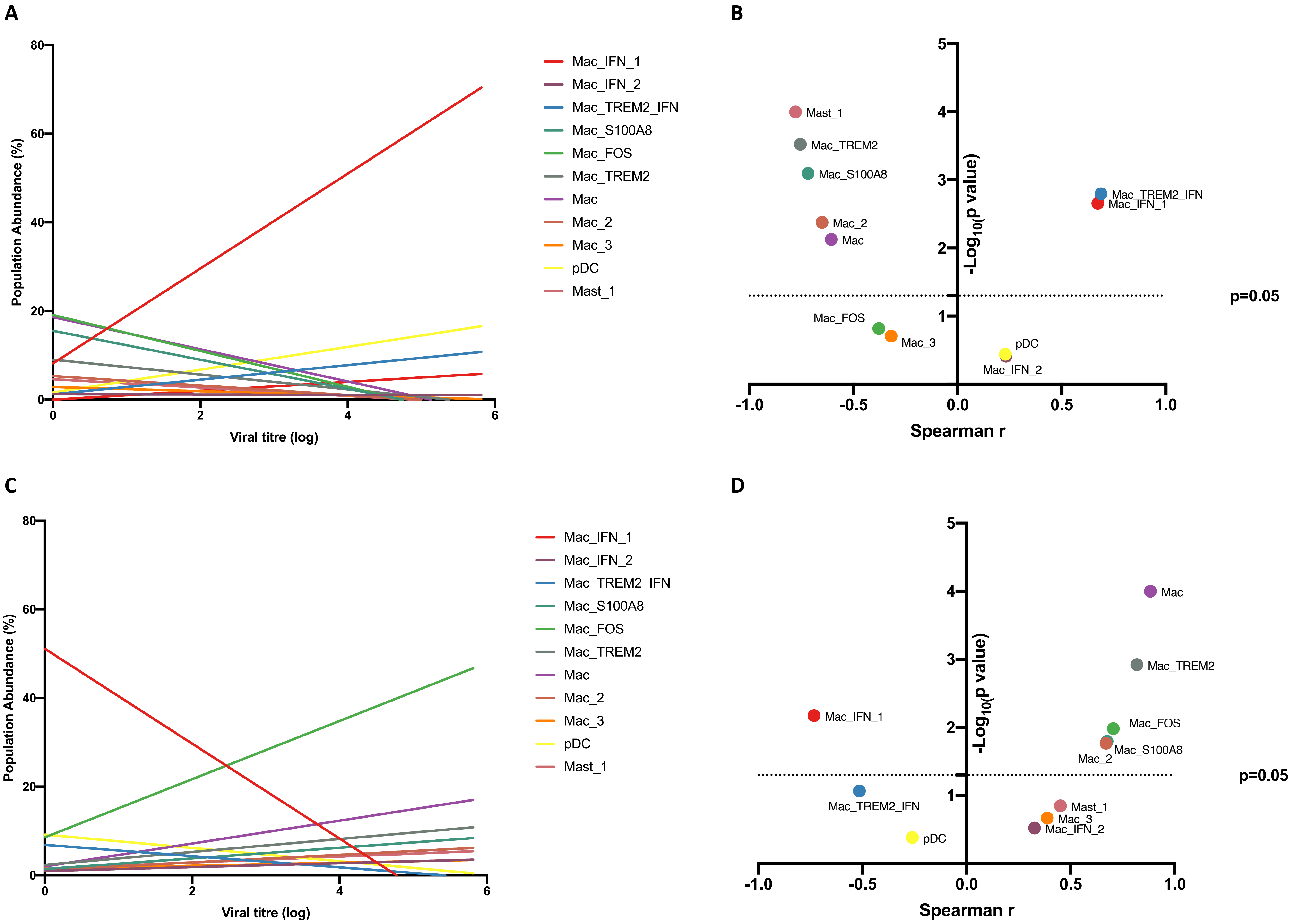

Supplementary Figure 23. (A) Correlation curves depicting trends between fraction of cellular subsets and Log10 viral titers in BAL at the same time point. (B) Plot depicting Spearman correlation coefficient and associated p values. (C) Correlation curves depicting trends between fraction of cellular subsets and Log10 viral titers in BAL at the subsequent time point. (D) Plot depicting Spearman correlation coefficient and associated p values.

Supplementary Table 1: List of animals and demographics.

Annotated ID	Species	Common name	Gender	Age (Year and Days)	Weight (kg)
MM09	Macaca mulatta	Rhesus monkey	Male	3 years, 350 days	6
MM10	Macaca mulatta	Rhesus monkey	Male	3 years, 343 days	6.5
MM11	Macaca mulatta	Rhesus monkey	Female	3 years, 323 days	5.84
MM12	Macaca mulatta	Rhesus monkey	Female	3 years, 324 days	4.44
MM14	Macaca mulatta	Rhesus monkey	Female	3 years, 269 days	5.7
MM15	Macaca mulatta	Rhesus monkey	Male	3 years, 258 days	5.72
MM13	Macaca mulatta	Rhesus monkey	Female	3 years, 316 days	4.21
MM16	Macaca mulatta	Rhesus monkey	Male	3 years, 19 days	4.81

Supplementary table 2: Number of cells analyzed for pan-CD45 clusters across the sampled timepoints.

Cluster_new	Cells_count
Mac_IFN	32984
Mac_IFN_2	1613
Mac_IFN_3	165
Mac_MARCO_1	27440
Mac_MARCO_2	27362
Mac_MARCO_3	6110
Mac_MARCO_4	5636
Mac_TREM2	7400
Mac_TREM2_IFN	4412
Mac_BIRC3	1413
pDC_1	5693
pDC_2	59
MAST_cells	3525
DC	2583
T_NK_1	27812
T_NK_2	6105
B_cells	3293
12	2885
Prolif	1904
non_imm_1	1217
non_imm_2	467

Supplementary table 3: Number of cells analyzed for pan-CD45 clusters across the sampled timepoints.

Subject	Condition	Cluster_new	Cells_count
35951	w-1	Mac_IFN	20
35951	w-1	Mac_IFN_3	32
35951	w-1	Mac_MARCO_1	205

35951	w-1	Mac_MARCO_2	3013
35951	w-1	Mac_MARCO_3	296
35951	w-1	Mac_MARCO_4	1
35951	w-1	Mac_TREM2	416
35951	w-1	Mac_TREM2_IFN	15
35951	w-1	Mac_BIRC3	18
35951	w-1	pDC_1	62
35951	w-1	pDC_2	2
35951	w-1	MAST_cells	565
35951	w-1	DC	36
35951	w-1	T_NK_1	1279
35951	w-1	T_NK_2	147
35951	w-1	B_cells	37
35951	w-1	12	49
35951	w-1	Prolif	20
35951	w-1	non_imm_1	5
35951	w-1	non_imm_2	20
35951	d3	Mac_IFN	2801
35951	d3	Mac_IFN_2	82
35951	d3	Mac_MARCO_1	4
35951	d3	Mac_MARCO_2	3
35951	d3	Mac_MARCO_3	2
35951	d3	Mac_MARCO_4	232
35951	d3	Mac_TREM2	11
35951	d3	Mac_TREM2_IFN	304
35951	d3	Mac_BIRC3	40
35951	d3	pDC_1	11
35951	d3	pDC_2	4
35951	d3	MAST_cells	450
35951	d3	DC	164
35951	d3	T_NK_1	685
35951	d3	T_NK_2	57
35951	d3	B_cells	38
35951	d3	12	98
35951	d3	Prolif	20
35951	d3	non_imm_1	17
35951	d3	non_imm_2	20
35951	necropsy	Mac_IFN	3
35951	necropsy	Mac_IFN_3	11
35951	necropsy	Mac_MARCO_1	1474
35951	necropsy	Mac_MARCO_2	31

35951	necropsy	Mac_MARCO_3	37
35951	necropsy	Mac_MARCO_4	414
35951	necropsy	Mac_TREM2	209
35951	necropsy	Mac_TREM2_IFN	21
35951	necropsy	Mac_BIRC3	28
35951	necropsy	pDC_1	39
35951	necropsy	pDC_2	10
35951	necropsy	MAST_cells	575
35951	necropsy	DC	92
35951	necropsy	T_NK_1	1200
35951	necropsy	T_NK_2	558
35951	necropsy	B_cells	345
35951	necropsy	12	112
35951	necropsy	Prolif	47
35951	necropsy	non_imm_1	13
35951	necropsy	non_imm_2	2
35969	w-1	Mac_IFN	283
35969	w-1	Mac_IFN_2	28
35969	w-1	Mac_MARCO_1	161
35969	w-1	Mac_MARCO_2	4680
35969	w-1	Mac_MARCO_3	1452
35969	w-1	Mac_MARCO_4	23
35969	w-1	Mac_TREM2	502
35969	w-1	Mac_TREM2_IFN	247
35969	w-1	Mac_BIRC3	34
35969	w-1	pDC_1	263
35969	w-1	pDC_2	3
35969	w-1	MAST_cells	131
35969	w-1	DC	83
35969	w-1	T_NK_1	1571
35969	w-1	T_NK_2	180
35969	w-1	B_cells	69
35969	w-1	12	151
35969	w-1	Prolif	113
35969	w-1	non_imm_1	86
35969	w-1	non_imm_2	76
35969	d3	Mac_IFN	6301
35969	d3	Mac_IFN_2	245
35969	d3	Mac_MARCO_1	28
35969	d3	Mac_MARCO_2	1293
35969	d3	Mac_MARCO_3	38

35969	d3	Mac_MARCO_4	75
35969	d3	Mac_TREM2	181
35969	d3	Mac_TREM2_IFN	94
35969	d3	Mac_BIRC3	12
35969	d3	pDC_1	88
35969	d3	pDC_2	2
35969	d3	MAST_cells	35
35969	d3	DC	24
35969	d3	T_NK_1	575
35969	d3	T_NK_2	47
35969	d3	B_cells	79
35969	d3	12	134
35969	d3	Prolif	109
35969	d3	non_imm_1	60
35969	d3	non_imm_2	11
35969	necropsy	Mac_IFN	28
35969	necropsy	Mac_MARCO_1	3877
35969	necropsy	Mac_MARCO_2	66
35969	necropsy	Mac_MARCO_3	37
35969	necropsy	Mac_MARCO_4	847
35969	necropsy	Mac_TREM2	408
35969	necropsy	Mac_TREM2_IFN	49
35969	necropsy	Mac_BIRC3	24
35969	necropsy	pDC_1	36
35969	necropsy	pDC_2	7
35969	necropsy	MAST_cells	150
35969	necropsy	DC	192
35969	necropsy	T_NK_1	1591
35969	necropsy	T_NK_2	155
35969	necropsy	B_cells	95
35969	necropsy	12	31
35969	necropsy	Prolif	122
35969	necropsy	non_imm_1	49
35969	necropsy	non_imm_2	1
36052	w-1	Mac_IFN	2
36052	w-1	Mac_IFN_3	67
36052	w-1	Mac_MARCO_1	112
36052	w-1	Mac_MARCO_2	4212
36052	w-1	Mac_MARCO_3	1380
36052	w-1	Mac_MARCO_4	37
36052	w-1	Mac_TREM2	309

36052	w-1	Mac_TREM2_IFN	49
36052	w-1	Mac_BIRC3	42
36052	w-1	pDC_1	27
36052	w-1	MAST_cells	146
36052	w-1	DC	64
36052	w-1	T_NK_1	3275
36052	w-1	T_NK_2	335
36052	w-1	B_cells	147
36052	w-1	12	75
36052	w-1	Prolif	101
36052	w-1	non_imm_1	216
36052	w-1	non_imm_2	50
36052	d3	Mac_IFN	11667
36052	d3	Mac_IFN_2	814
36052	d3	Mac_MARCO_1	6
36052	d3	Mac_MARCO_2	17
36052	d3	Mac_MARCO_3	2
36052	d3	Mac_MARCO_4	172
36052	d3	Mac_TREM2	50
36052	d3	Mac_TREM2_IFN	583
36052	d3	Mac_BIRC3	37
36052	d3	pDC_1	105
36052	d3	MAST_cells	154
36052	d3	DC	161
36052	d3	T_NK_1	1859
36052	d3	T_NK_2	271
36052	d3	B_cells	109
36052	d3	12	87
36052	d3	Prolif	202
36052	d3	non_imm_1	194
36052	d3	non_imm_2	118
36052	necropsy	Mac_IFN	12
36052	necropsy	Mac_IFN_2	1
36052	necropsy	Mac_IFN_3	55
36052	necropsy	Mac_MARCO_1	7268
36052	necropsy	Mac_MARCO_2	1960
36052	necropsy	Mac_MARCO_3	39
36052	necropsy	Mac_MARCO_4	697
36052	necropsy	Mac_TREM2	894
36052	necropsy	Mac_TREM2_IFN	130
36052	necropsy	Mac_BIRC3	92

36052	necropsy	pDC_1	77
36052	necropsy	MAST_cells	258
36052	necropsy	DC	319
36052	necropsy	T_NK_1	1569
36052	necropsy	T_NK_2	460
36052	necropsy	B_cells	442
36052	necropsy	12	144
36052	necropsy	Prolif	188
36052	necropsy	non_imm_1	34
36052	necropsy	non_imm_2	11
36056	w-1	Mac_IFN	738
36056	w-1	Mac_IFN_2	4
36056	w-1	Mac_MARCO_1	44
36056	w-1	Mac_MARCO_2	5036
36056	w-1	Mac_MARCO_3	806
36056	w-1	Mac_MARCO_4	3
36056	w-1	Mac_TREM2	927
36056	w-1	Mac_TREM2_IFN	15
36056	w-1	Mac_BIRC3	62
36056	w-1	pDC_1	48
36056	w-1	MAST_cells	254
36056	w-1	DC	179
36056	w-1	T_NK_1	2219
36056	w-1	T_NK_2	515
36056	w-1	B_cells	212
36056	w-1	12	52
36056	w-1	Prolif	174
36056	w-1	non_imm_1	88
36056	w-1	non_imm_2	36
36056	d3	Mac_IFN	1094
36056	d3	Mac_IFN_2	104
36056	d3	Mac_MARCO_1	33
36056	d3	Mac_MARCO_2	3
36056	d3	Mac_MARCO_3	6
36056	d3	Mac_MARCO_4	130
36056	d3	Mac_TREM2	43
36056	d3	Mac_TREM2_IFN	1844
36056	d3	Mac_BIRC3	475
36056	d3	pDC_1	4142
36056	d3	MAST_cells	26
36056	d3	DC	213

36056	d3	T_NK_1	1536
36056	d3	T_NK_2	694
36056	d3	B_cells	412
36056	d3	12	283
36056	d3	Prolif	83
36056	d3	non_imm_1	30
36056	d3	non_imm_2	20
36056	necropsy	Mac_IFN	1218
36056	necropsy	Mac_IFN_2	16
36056	necropsy	Mac_MARCO_1	5319
36056	necropsy	Mac_MARCO_2	92
36056	necropsy	Mac_MARCO_3	121
36056	necropsy	Mac_MARCO_4	1140
36056	necropsy	Mac_TREM2	800
36056	necropsy	Mac_TREM2_IFN	23
36056	necropsy	Mac_BIRC3	31
36056	necropsy	pDC_1	21
36056	necropsy	MAST_cells	62
36056	necropsy	DC	311
36056	necropsy	T_NK_1	2277
36056	necropsy	T_NK_2	492
36056	necropsy	B_cells	105
36056	necropsy	12	1209
36056	necropsy	Prolif	163
36056	necropsy	non_imm_1	15
36056	necropsy	non_imm_2	6
36189	w-1	Mac_IFN	55
36189	w-1	Mac_IFN_2	5
36189	w-1	Mac_MARCO_1	12
36189	w-1	Mac_MARCO_2	2428
36189	w-1	Mac_MARCO_3	861
36189	w-1	Mac_MARCO_4	3
36189	w-1	Mac_TREM2	347
36189	w-1	Mac_TREM2_IFN	8
36189	w-1	Mac_BIRC3	21
36189	w-1	pDC_1	33
36189	w-1	pDC_2	6
36189	w-1	MAST_cells	282
36189	w-1	DC	66
36189	w-1	T_NK_1	1656
36189	w-1	T_NK_2	372

36189	w-1	B_cells	276
36189	w-1	12	56
36189	w-1	Prolif	63
36189	w-1	non_imm_1	43
36189	w-1	non_imm_2	12
36189	d3	Mac_IFN	3710
36189	d3	Mac_IFN_2	164
36189	d3	Mac_MARCO_1	31
36189	d3	Mac_MARCO_2	14
36189	d3	Mac_MARCO_3	14
36189	d3	Mac_MARCO_4	863
36189	d3	Mac_TREM2	118
36189	d3	Mac_TREM2_IFN	413
36189	d3	Mac_BIRC3	21
36189	d3	pDC_1	14
36189	d3	pDC_2	25
36189	d3	MAST_cells	97
36189	d3	DC	217
36189	d3	T_NK_1	1720
36189	d3	T_NK_2	373
36189	d3	B_cells	76
36189	d3	12	75
36189	d3	Prolif	73
36189	d3	non_imm_1	8
36189	d3	non_imm_2	4
36189	necropsy	Mac_IFN	390
36189	necropsy	Mac_MARCO_1	6100
36189	necropsy	Mac_MARCO_2	1264
36189	necropsy	Mac_MARCO_3	6
36189	necropsy	Mac_MARCO_4	249
36189	necropsy	Mac_TREM2	922
36189	necropsy	Mac_TREM2_IFN	158
36189	necropsy	Mac_BIRC3	116
36189	necropsy	pDC_1	136
36189	necropsy	MAST_cells	283
36189	necropsy	DC	218
36189	necropsy	T_NK_1	750
36189	necropsy	T_NK_2	180
36189	necropsy	B_cells	218
36189	necropsy	12	162
36189	necropsy	Prolif	198

36189	necropsy	non_imm_1	3
36189	necropsy	non_imm_2	1
36207	w-1	Mac_IFN	135
36207	w-1	Mac_IFN_2	4
36207	w-1	Mac_MARCO_1	103
36207	w-1	Mac_MARCO_2	2486
36207	w-1	Mac_MARCO_3	925
36207	w-1	Mac_MARCO_4	22
36207	w-1	Mac_TREM2	571
36207	w-1	Mac_TREM2_IFN	6
36207	w-1	Mac_BIRC3	28
36207	w-1	pDC_1	7
36207	w-1	MAST_cells	32
36207	w-1	DC	45
36207	w-1	T_NK_1	785
36207	w-1	T_NK_2	195
36207	w-1	B_cells	172
36207	w-1	12	46
36207	w-1	Prolif	65
36207	w-1	non_imm_1	193
36207	w-1	non_imm_2	48
36207	d3	Mac_IFN	4496
36207	d3	Mac_IFN_2	146
36207	d3	Mac_MARCO_1	20
36207	d3	Mac_MARCO_2	704
36207	d3	Mac_MARCO_3	48
36207	d3	Mac_MARCO_4	42
36207	d3	Mac_TREM2	205
36207	d3	Mac_TREM2_IFN	443
36207	d3	Mac_BIRC3	294
36207	d3	pDC_1	567
36207	d3	MAST_cells	7
36207	d3	DC	47
36207	d3	T_NK_1	898
36207	d3	T_NK_2	273
36207	d3	B_cells	190
36207	d3	12	103
36207	d3	Prolif	85
36207	d3	non_imm_1	160
36207	d3	non_imm_2	31
36207	necropsy	Mac_IFN	31

36207	necropsy	Mac_MARCO_1	2643
36207	necropsy	Mac_MARCO_2	60
36207	necropsy	Mac_MARCO_3	40
36207	necropsy	Mac_MARCO_4	686
36207	necropsy	Mac_TREM2	487
36207	necropsy	Mac_TREM2_IFN	10
36207	necropsy	Mac_BIRC3	38
36207	necropsy	pDC_1	17
36207	necropsy	MAST_cells	18
36207	necropsy	DC	152
36207	necropsy	T_NK_1	2367
36207	necropsy	T_NK_2	801
36207	necropsy	B_cells	271
36207	necropsy	12	18
36207	necropsy	Prolif	78
36207	necropsy	non_imm_1	3

Supplementary table 4: Number of cells analyzed for myeloid clusters.

Cluster_new	Cells_count
Mac_IFN_1	31190
Mac_IFN_2	1706
Mac_TREM2_IFN	4463
Mac_S100A8	15225
Mac_FOS	15070
Mac_TREM2	8471
Mac	18196
Mac_2	4708
Mac_3	2893
Mac_dead	3300
pDC	6720
cDC2_and_doubl	2806
cDC1_and_BIRC3	2040
Mast_1	3152
Mast_2	573
Mac_T_doublet	6764
Mac_IFN_T_doublet	2003

Supplementary table 5: Number of cells analyzed for myeloid clusters across the sampled timepoints.

Animal	Condition	Cluster_new	Cells_count
35951	d3	Mac_IFN_1	2781
35951	d3	Mac_IFN_2	9

35951	d3	Mac_TREM2_IFN	328
35951	d3	Mac_S100A8	4
35951	d3	Mac_TREM2	11
35951	d3	Mac	8
35951	d3	Mac_2	1
35951	d3	Mac_3	50
35951	d3	Mac_dead	201
35951	d3	pDC	9
35951	d3	cDC2_and_doubl	167
35951	d3	cDC1_and_BIRC3	48
35951	d3	Mast_1	20
35951	d3	Mast_2	429
35951	d3	Mac_T_doublet	35
35951	d3	Mac_IFN_T_doublet	105
35951	necropsy	Mac_TREM2_IFN	46
35951	necropsy	Mac_S100A8	650
35951	necropsy	Mac_FOS	1
35951	necropsy	Mac_TREM2	276
35951	necropsy	Mac	848
35951	necropsy	Mac_2	208
35951	necropsy	Mac_3	60
35951	necropsy	Mac_dead	61
35951	necropsy	pDC	101
35951	necropsy	cDC2_and_doubl	55
35951	necropsy	cDC1_and_BIRC3	75
35951	necropsy	Mast_1	577
35951	necropsy	Mac_T_doublet	98
35951	w-1	Mac_IFN_1	7
35951	w-1	Mac_IFN_2	66
35951	w-1	Mac_TREM2_IFN	41
35951	w-1	Mac_S100A8	379
35951	w-1	Mac_FOS	1871
35951	w-1	Mac_TREM2	466
35951	w-1	Mac	620
35951	w-1	Mac_2	176
35951	w-1	Mac_3	65
35951	w-1	Mac_dead	86
35951	w-1	pDC	143
35951	w-1	cDC2_and_doubl	43
35951	w-1	cDC1_and_BIRC3	31
35951	w-1	Mast_1	561

35951	w-1	Mac_T_doublet	175
35969	d3	Mac_IFN_1	6113
35969	d3	Mac_IFN_2	255
35969	d3	Mac_TREM2_IFN	171
35969	d3	Mac_S100A8	107
35969	d3	Mac_FOS	175
35969	d3	Mac_TREM2	182
35969	d3	Mac	547
35969	d3	Mac_2	132
35969	d3	Mac_3	47
35969	d3	Mac_dead	324
35969	d3	pDC	169
35969	d3	cDC2_and_doubl	41
35969	d3	cDC1_and_BIRC3	18
35969	d3	Mast_1	32
35969	d3	Mac_T_doublet	77
35969	d3	Mac_IFN_T_doublet	160
35969	necropsy	Mac_IFN_1	39
35969	necropsy	Mac_TREM2_IFN	28
35969	necropsy	Mac_S100A8	1864
35969	necropsy	Mac_FOS	26
35969	necropsy	Mac_TREM2	587
35969	necropsy	Mac	1788
35969	necropsy	Mac_2	484
35969	necropsy	Mac_3	38
35969	necropsy	Mac_dead	124
35969	necropsy	pDC	85
35969	necropsy	cDC2_and_doubl	222
35969	necropsy	cDC1_and_BIRC3	55
35969	necropsy	Mast_1	149
35969	necropsy	Mac_T_doublet	261
35969	necropsy	Mac_IFN_T_doublet	2
35969	w-1	Mac_IFN_1	251
35969	w-1	Mac_IFN_2	7
35969	w-1	Mac_TREM2_IFN	240
35969	w-1	Mac_S100A8	965
35969	w-1	Mac_FOS	2562
35969	w-1	Mac_TREM2	581
35969	w-1	Mac	1338
35969	w-1	Mac_2	333
35969	w-1	Mac_3	370

35969	w-1	Mac_dead	122
35969	w-1	pDC	516
35969	w-1	cDC2_and_doubl	127
35969	w-1	cDC1_and_BIRC3	56
35969	w-1	Mast_1	125
35969	w-1	Mac_T_doublet	436
35969	w-1	Mac_IFN_T_doublet	12
36052	d3	Mac_IFN_1	10622
36052	d3	Mac_IFN_2	234
36052	d3	Mac_TREM2_IFN	640
36052	d3	Mac_S100A8	1
36052	d3	Mac_TREM2	33
36052	d3	Mac	10
36052	d3	Mac_3	7
36052	d3	Mac_dead	611
36052	d3	pDC	104
36052	d3	cDC2_and_doubl	179
36052	d3	cDC1_and_BIRC3	54
36052	d3	Mast_1	15
36052	d3	Mast_2	138
36052	d3	Mac_T_doublet	176
36052	d3	Mac_IFN_T_doublet	1031
36052	necropsy	Mac_IFN_1	3
36052	necropsy	Mac_IFN_2	2
36052	necropsy	Mac_TREM2_IFN	135
36052	necropsy	Mac_S100A8	3008
36052	necropsy	Mac_FOS	259
36052	necropsy	Mac_TREM2	1021
36052	necropsy	Mac	3692
36052	necropsy	Mac_2	742
36052	necropsy	Mac_3	81
36052	necropsy	Mac_dead	343
36052	necropsy	pDC	223
36052	necropsy	cDC2_and_doubl	356
36052	necropsy	cDC1_and_BIRC3	174
36052	necropsy	Mast_1	254
36052	necropsy	Mac_T_doublet	1646
36052	necropsy	Mac_IFN_T_doublet	7
36052	w-1	Mac_IFN_2	20
36052	w-1	Mac_TREM2_IFN	112
36052	w-1	Mac_S100A8	460

36052	w-1	Mac_FOS	3350
36052	w-1	Mac_TREM2	293
36052	w-1	Mac	470
36052	w-1	Mac_2	140
36052	w-1	Mac_3	325
36052	w-1	Mac_dead	131
36052	w-1	pDC	189
36052	w-1	cDC2_and_doubl	115
36052	w-1	cDC1_and_BIRC3	61
36052	w-1	Mast_1	146
36052	w-1	Mac_T_doublet	706
36052	w-1	Mac_IFN_T_doublet	4
36056	d3	Mac_IFN_1	1353
36056	d3	Mac_IFN_2	5
36056	d3	Mac_TREM2_IFN	1749
36056	d3	Mac_S100A8	18
36056	d3	Mac_FOS	1
36056	d3	Mac_TREM2	64
36056	d3	Mac	8
36056	d3	Mac_2	2
36056	d3	Mac_3	58
36056	d3	Mac_dead	95
36056	d3	pDC	4112
36056	d3	cDC2_and_doubl	104
36056	d3	cDC1_and_BIRC3	595
36056	d3	Mast_1	19
36056	d3	Mast_2	6
36056	d3	Mac_T_doublet	26
36056	d3	Mac_IFN_T_doublet	181
36056	necropsy	Mac_IFN_1	905
36056	necropsy	Mac_IFN_2	1
36056	necropsy	Mac_TREM2_IFN	17
36056	necropsy	Mac_S100A8	3502
36056	necropsy	Mac_FOS	13
36056	necropsy	Mac_TREM2	1362
36056	necropsy	Mac	1571
36056	necropsy	Mac_2	103
36056	necropsy	Mac_3	1135
36056	necropsy	Mac_dead	198
36056	necropsy	pDC	21
36056	necropsy	cDC2_and_doubl	305

36056	necropsy	cDC1_and_BIRC3	70
36056	necropsy	Mast_1	62
36056	necropsy	Mac_T_doublet	1056
36056	necropsy	Mac_IFN_T_doublet	42
36056	w-1	Mac_IFN_1	109
36056	w-1	Mac_IFN_2	939
36056	w-1	Mac_TREM2_IFN	15
36056	w-1	Mac_S100A8	26
36056	w-1	Mac_FOS	2932
36056	w-1	Mac_TREM2	802
36056	w-1	Mac	1468
36056	w-1	Mac_2	384
36056	w-1	Mac_3	28
36056	w-1	Mac_dead	88
36056	w-1	pDC	47
36056	w-1	cDC2_and_doubl	293
36056	w-1	cDC1_and_BIRC3	118
36056	w-1	Mast_1	254
36056	w-1	Mac_T_doublet	663
36056	w-1	Mac_IFN_T_doublet	2
36189	d3	Mac_IFN_1	4015
36189	d3	Mac_IFN_2	8
36189	d3	Mac_TREM2_IFN	391
36189	d3	Mac_S100A8	108
36189	d3	Mac_FOS	1
36189	d3	Mac_TREM2	178
36189	d3	Mac	3
36189	d3	Mac_2	27
36189	d3	Mac_3	52
36189	d3	Mac_dead	344
36189	d3	pDC	48
36189	d3	cDC2_and_doubl	190
36189	d3	cDC1_and_BIRC3	64
36189	d3	Mast_1	97
36189	d3	Mac_T_doublet	18
36189	d3	Mac_IFN_T_doublet	232
36189	necropsy	Mac_IFN_1	63
36189	necropsy	Mac_IFN_2	1
36189	necropsy	Mac_TREM2_IFN	89
36189	necropsy	Mac_S100A8	2469
36189	necropsy	Mac_FOS	37

36189	necropsy	Mac_TREM2	981
36189	necropsy	Mac	3490
36189	necropsy	Mac_2	969
36189	necropsy	Mac_3	73
36189	necropsy	Mac_dead	227
36189	necropsy	pDC	252
36189	necropsy	cDC2_and_doubl	243
36189	necropsy	cDC1_and_BIRC3	177
36189	necropsy	Mast_1	250
36189	necropsy	Mac_T_doublet	679
36189	necropsy	Mac_IFN_T_doublet	4
36189	w-1	Mac_IFN_1	42
36189	w-1	Mac_IFN_2	76
36189	w-1	Mac_TREM2_IFN	5
36189	w-1	Mac_S100A8	143
36189	w-1	Mac_FOS	2162
36189	w-1	Mac_TREM2	332
36189	w-1	Mac	118
36189	w-1	Mac_2	290
36189	w-1	Mac_3	235
36189	w-1	Mac_dead	52
36189	w-1	pDC	137
36189	w-1	cDC2_and_doubl	72
36189	w-1	cDC1_and_BIRC3	40
36189	w-1	Mast_1	282
36189	w-1	Mac_T_doublet	196
36189	w-1	Mac_IFN_T_doublet	1
36207	d3	Mac_IFN_1	4697
36207	d3	Mac_IFN_2	63
36207	d3	Mac_TREM2_IFN	439
36207	d3	Mac_S100A8	47
36207	d3	Mac_FOS	20
36207	d3	Mac_TREM2	187
36207	d3	Mac	213
36207	d3	Mac_2	38
36207	d3	Mac_3	65
36207	d3	Mac_dead	185
36207	d3	pDC	538
36207	d3	cDC2_and_doubl	45
36207	d3	cDC1_and_BIRC3	304
36207	d3	Mast_1	15

36207	d3	Mac_T_doublet	48
36207	d3	Mac_IFN_T_doublet	218
36207	necropsy	Mac_IFN_1	47
36207	necropsy	Mac_IFN_2	2
36207	necropsy	Mac_TREM2_IFN	11
36207	necropsy	Mac_S100A8	1081
36207	necropsy	Mac_FOS	54
36207	necropsy	Mac_TREM2	568
36207	necropsy	Mac	1382
36207	necropsy	Mac_2	271
36207	necropsy	Mac_3	21
36207	necropsy	Mac_dead	61
36207	necropsy	pDC	17
36207	necropsy	cDC2_and_doubl	191
36207	necropsy	cDC1_and_BIRC3	60
36207	necropsy	Mast_1	80
36207	necropsy	Mac_T_doublet	353
36207	necropsy	Mac_IFN_T_doublet	1
36207	w-1	Mac_IFN_1	143
36207	w-1	Mac_IFN_2	18
36207	w-1	Mac_TREM2_IFN	6
36207	w-1	Mac_S100A8	393
36207	w-1	Mac_FOS	1606
36207	w-1	Mac_TREM2	547
36207	w-1	Mac	622
36207	w-1	Mac_2	408
36207	w-1	Mac_3	183
36207	w-1	Mac_dead	47
36207	w-1	pDC	9
36207	w-1	cDC2_and_doubl	58
36207	w-1	cDC1_and_BIRC3	40
36207	w-1	Mast_1	214
36207	w-1	Mac_T_doublet	115
36207	w-1	Mac_IFN_T_doublet	1

Supplementary table 6: Number of cells analyzed for lymphoid clusters.

Cluster_new	Cells_count
T_1	8331
T_2	6733
T_3	4914
T_4	4206

T_5	753
T_IFN	2682
NK_Tgd	5203
Treg	412
B_cells_1	2823
B_cells_2	142
B_cells_3	107
T_doublet_1	1493
T_doublet_2	361

Supplementary table 7: Number of cells analyzed for lymphoid clusters across the sampled timepoints.

Animal	Condition	Cluster_new	Cells_count
35951	d3	T_1	2
35951	d3	T_2	8
35951	d3	T_3	2
35951	d3	T_4	536
35951	d3	T_5	4
35951	d3	T_IFN	94
35951	d3	NK_Tgd	78
35951	d3	Treg	1
35951	d3	B_cells_1	8
35951	d3	B_cells_2	1
35951	d3	B_cells_3	29
35951	d3	T_doublet_1	86
35951	d3	T_doublet_2	1
35951	necropsy	T_1	145
35951	necropsy	T_2	531
35951	necropsy	T_3	481
35951	necropsy	T_4	5
35951	necropsy	T_IFN	61
35951	necropsy	NK_Tgd	522
35951	necropsy	Treg	27
35951	necropsy	B_cells_1	339
35951	necropsy	B_cells_2	3
35951	necropsy	T_doublet_1	58
35951	necropsy	T_doublet_2	5
35951	w-1	T_1	829
35951	w-1	T_2	72
35951	w-1	T_3	206
35951	w-1	T_4	1

35951	w-1	T_IFN	29
35951	w-1	NK_Tgd	108
35951	w-1	Treg	23
35951	w-1	B_cells_1	37
35951	w-1	T_doublet_1	136
35951	w-1	T_doublet_2	38
35969	d3	T_1	392
35969	d3	T_2	3
35969	d3	T_3	23
35969	d3	T_4	34
35969	d3	T_5	1
35969	d3	T_IFN	105
35969	d3	NK_Tgd	37
35969	d3	Treg	7
35969	d3	B_cells_1	68
35969	d3	B_cells_2	3
35969	d3	T_doublet_1	91
35969	necropsy	T_1	24
35969	necropsy	T_2	777
35969	necropsy	T_3	446
35969	necropsy	T_4	200
35969	necropsy	T_5	49
35969	necropsy	T_IFN	31
35969	necropsy	NK_Tgd	161
35969	necropsy	Treg	26
35969	necropsy	B_cells_1	85
35969	necropsy	B_cells_2	2
35969	necropsy	T_doublet_1	108
35969	necropsy	T_doublet_2	2
35969	w-1	T_1	927
35969	w-1	T_2	140
35969	w-1	T_3	161
35969	w-1	T_4	187
35969	w-1	T_5	34
35969	w-1	T_IFN	59
35969	w-1	NK_Tgd	138
35969	w-1	Treg	19
35969	w-1	B_cells_1	64
35969	w-1	B_cells_2	10
35969	w-1	T_doublet_1	105
35969	w-1	T_doublet_2	1

36052	d3	T_1	16
36052	d3	T_2	31
36052	d3	T_3	29
36052	d3	T_4	1481
36052	d3	T_5	274
36052	d3	T_IFN	203
36052	d3	NK_Tgd	226
36052	d3	Treg	1
36052	d3	B_cells_1	7
36052	d3	B_cells_2	4
36052	d3	B_cells_3	78
36052	d3	T_doublet_1	30
36052	d3	T_doublet_2	6
36052	necropsy	T_1	47
36052	necropsy	T_2	950
36052	necropsy	T_3	421
36052	necropsy	T_5	2
36052	necropsy	T_IFN	81
36052	necropsy	NK_Tgd	464
36052	necropsy	Treg	46
36052	necropsy	B_cells_1	352
36052	necropsy	B_cells_2	17
36052	necropsy	T_doublet_1	204
36052	necropsy	T_doublet_2	7
36052	w-1	T_1	1944
36052	w-1	T_2	581
36052	w-1	T_3	559
36052	w-1	T_5	1
36052	w-1	T_IFN	85
36052	w-1	NK_Tgd	299
36052	w-1	Treg	26
36052	w-1	B_cells_1	135
36052	w-1	B_cells_2	2
36052	w-1	T_doublet_1	55
36052	w-1	T_doublet_2	95
36056	d3	T_1	158
36056	d3	T_2	107
36056	d3	T_3	149
36056	d3	T_4	207
36056	d3	T_5	8
36056	d3	T_IFN	1039

36056	d3	NK_Tgd	498
36056	d3	Treg	11
36056	d3	B_cells_1	342
36056	d3	B_cells_2	62
36056	d3	T_doublet_1	36
36056	d3	T_doublet_2	12
36056	necropsy	T_1	285
36056	necropsy	T_2	445
36056	necropsy	T_3	807
36056	necropsy	T_4	503
36056	necropsy	T_5	157
36056	necropsy	T_IFN	8
36056	necropsy	NK_Tgd	356
36056	necropsy	Treg	24
36056	necropsy	B_cells_1	82
36056	necropsy	B_cells_2	4
36056	necropsy	T_doublet_1	167
36056	necropsy	T_doublet_2	63
36056	w-1	T_1	1269
36056	w-1	T_2	542
36056	w-1	T_3	222
36056	w-1	T_4	4
36056	w-1	T_5	1
36056	w-1	T_IFN	53
36056	w-1	NK_Tgd	521
36056	w-1	Treg	35
36056	w-1	B_cells_1	190
36056	w-1	B_cells_2	6
36056	w-1	T_doublet_1	47
36056	w-1	T_doublet_2	70
36189	d3	T_1	105
36189	d3	T_2	623
36189	d3	T_3	438
36189	d3	T_4	264
36189	d3	T_5	41
36189	d3	T_IFN	283
36189	d3	NK_Tgd	309
36189	d3	Treg	18
36189	d3	B_cells_1	71
36189	d3	B_cells_2	1
36189	d3	T_doublet_1	44

36189	d3	T_doublet_2	1
36189	necropsy	T_1	96
36189	necropsy	T_2	371
36189	necropsy	T_3	96
36189	necropsy	T_4	116
36189	necropsy	T_5	31
36189	necropsy	T_IFN	24
36189	necropsy	NK_Tgd	150
36189	necropsy	Treg	37
36189	necropsy	B_cells_1	183
36189	necropsy	B_cells_2	11
36189	necropsy	T_doublet_1	59
36189	necropsy	T_doublet_2	21
36189	w-1	T_1	1044
36189	w-1	T_2	177
36189	w-1	T_3	250
36189	w-1	T_4	103
36189	w-1	T_5	27
36189	w-1	T_IFN	10
36189	w-1	NK_Tgd	362
36189	w-1	Treg	43
36189	w-1	B_cells_1	257
36189	w-1	B_cells_2	5
36189	w-1	T_doublet_1	31
36189	w-1	T_doublet_2	37
36207	d3	T_1	375
36207	d3	T_2	8
36207	d3	T_3	27
36207	d3	T_4	66
36207	d3	T_5	2
36207	d3	T_IFN	493
36207	d3	NK_Tgd	125
36207	d3	Treg	13
36207	d3	B_cells_1	176
36207	d3	B_cells_2	6
36207	d3	T_doublet_1	126
36207	necropsy	T_1	29
36207	necropsy	T_2	1325
36207	necropsy	T_3	546
36207	necropsy	T_4	467
36207	necropsy	T_5	117

36207	necropsy	T_IFN	15
36207	necropsy	NK_Tgd	684
36207	necropsy	Treg	43
36207	necropsy	B_cells_1	268
36207	necropsy	B_cells_2	5
36207	necropsy	T_doublet_1	44
36207	necropsy	T_doublet_2	1
36207	w-1	T_1	644
36207	w-1	T_2	42
36207	w-1	T_3	51
36207	w-1	T_4	32
36207	w-1	T_5	4
36207	w-1	T_IFN	9
36207	w-1	NK_Tgd	165
36207	w-1	Treg	12
36207	w-1	B_cells_1	159
36207	w-1	T_doublet_1	66
36207	w-1	T_doublet_2	1

Supplementary table 8: Details of antibodies.

Antibody	Dilution	Supplier	Catalogue Number	Clone	Validation Statement: Reactivity
ACE-2	1: 50	R & D Systems	AF933	Polyclonal	Human, Mouse, Rat, Hamster, in-house validation for NHPs
MX-1	1: 200	Thermo Fisher Scientific	PA5-22101	Polyclonal	Bovine, Human, Non-human primate, Rabbit
MX-2	1: 100	Millipore Sigma	HPA030235	Polyclonal	Human, in-house validation for NHPs
ISG-15	1: 100	Thermo Fisher Scientific	PA5-31865	Polyclonal	Human, Rat, in-house validation for NHPs
Siglec-1	1: 40	Novus Biologicals	NB600-534	HSn 7D2	Human, Mouse, Porcine, in-house validation for NHPs
SARS CoV2-Nucleocapsid	1: 100	Sino Biological	40588-T62	Polyclonal	SARS-CoV-2 Coronavirus Nucleocapsid
IFN-α (FITC Conjugated)	1: 10	PBL Assay Science	21112-3	MMHA-11	Human, in-house validation for NHPs
CD68	1: 40	Thermo Fisher Scientific	MA5-13324	KP1	Hamster, Human, Mouse, Pig, Rabbit, Rat, Rhesus monkey, Rat

HLA-DR	1: 40	Thermo Fisher Scientific	MA5-11966	LN3	Human, Mouse, Non-human primate, Rhesus monkey
CD123	1: 100	Thermo Fisher Scientific	PA5-85146	Polyclonal	Human, in-house validation for NHPs
CD3	1: 80	Agilent Technologies	A045201-2	Polyclonal	in-house validation for NHPs
C1q (FITC Conjugated)	1: 10	Abcam	ab4233	Polyclonal	Human, in-house validation for NHPs
**Secondary antibodies**					
Goat anti Mouse IgG, Alexa Fluor 647	1: 400	Thermo Fisher Scientific	A21236		Validated by manufacturer
Donkey anti Goat IgG, Alexa Fluor 555	1: 400	Thermo Fisher Scientific	A21432		Validated by manufacturer
Goat anti Mouse IgG, Alexa Fluor 555	1: 400	Thermo Fisher Scientific	A21422		Validated by manufacturer
Goat anti-rabbit IgG, Alexa Fluor 488	1: 400	Thermo Fisher Scientific	A11008		Validated by manufacturer
Goat anti-Mouse IgG, Alexa Fluor Plus 488	1: 400	Thermo Fisher Scientific	A32723		Validated by manufacturer
