# Supplementary material for: Myeloid cell interferon responses correlate with clearance of SARS-CoV-2
Source: Nat Commun. 2022 Feb 3;13:679. doi: 10.1038/s41467-022-28315-7 (PMC8814034; doi:10.1038/s41467-022-28315-7)
Supplement: Supplementary file 3 — Description of Additional Supplementary Information [file 41467_2022_28315_MOESM3_ESM.pdf]

Description of Additional Supplementary Files

File Name: Supplementary Data 1
Description: List of significantly perturbed genes across pan-CD45 clusters. Markers for each cluster were identified using Wilcoxon rank sum test and p-values were adjusted based using Bonferroni correction. No rounding operators were applied. Exact p-values and adjusted p-values are provided.

File Name: Supplementary Data 2
Description: List of significantly perturbed genes and their abundance across myeloid clusters. Markers for each cluster were identified using Wilcoxon rank sum test and p-values were adjusted based using Bonferroni correction. No rounding operators were applied. Exact p-values and adjusted p-values are provided.

File Name: Supplementary Data 3
Description: List of significantly perturbed genes and their abundance across lymphoid clusters. Markers for each cluster were identified using Wilcoxon rank sum test and p-values were adjusted based using Bonferroni correction. No rounding operators were applied. Exact p-values and adjusted p-values are provided.
